# Heat stress promotes Arabidopsis AGO1 phase separation and association with stress granule components

**DOI:** 10.1016/j.isci.2024.109151

**Published:** 2024-02-06

**Authors:** Aleksandar Blagojevic, Patricia Baldrich, Marlene Schiaffini, Esther Lechner, Nicolas Baumberger, Philippe Hammann, Taline Elmayan, Damien Garcia, Hervé Vaucheret, Blake C. Meyers, Pascal Genschik

**Affiliations:** 1 Institut de Biologie Moléculaire des Plantes, CNRS, Université de Strasbourg, 12, rue du Général Zimmer, 67084 Strasbourg, France; 2 Donald Danforth Plant Science Center, Saint Louis, MO 63132, USA; 3 Plateforme Protéomique Strasbourg Esplanade du CNRS, Université de Strasbourg, 67084 Strasbourg, France; 4 Université Paris-Saclay, INRAE, AgroParisTech, Institut Jean-Pierre Bourgin (IJPB), 78000 Versailles, France; 5 Division of Plant Science and Technology, University of Missouri, Columbia, MO 65211, USA

**Keywords:** Molecular biology, Cell biology, Plant biology

## Abstract

In *Arabidopsis thaliana*, ARGONAUTE1 (AGO1) plays a central role in microRNA (miRNA) and small interfering RNA (siRNA)-mediated silencing. AGO1 associates to the rough endoplasmic reticulum to conduct miRNA-mediated translational repression, mRNA cleavage, and biogenesis of phased siRNAs. Here, we show that a 37°C heat stress (HS) promotes AGO1 protein accumulation in cytosolic condensates where it colocalizes with components of siRNA bodies and of stress granules. AGO1 contains a prion-like domain in its poorly characterized N-terminal Poly-Q domain, which is sufficient to undergo phase separation independently of the presence of SGS3. HS only moderately affects the small RNA repertoire, the loading of AGO1 by miRNAs, and the signatures of target cleavage, suggesting that its localization in condensates protects AGO1 rather than promoting or impairing its activity in reprogramming gene expression during stress. Collectively, our work sheds new light on the impact of high temperature on a main effector of RNA silencing in plants.

## Highlights

• AGO1 dynamically localizes in SG components during HS without the requirement of SGS3
• Some P-bodies and RNA decay components are also part of the HS-induced AGO1 interactome
• A short HS has only limited effects on sRNA accumulation and their loading into AGO1
• AGO1 undergoes LLPS though its N-terminal domain

## Graphical abstract

**Figure:**
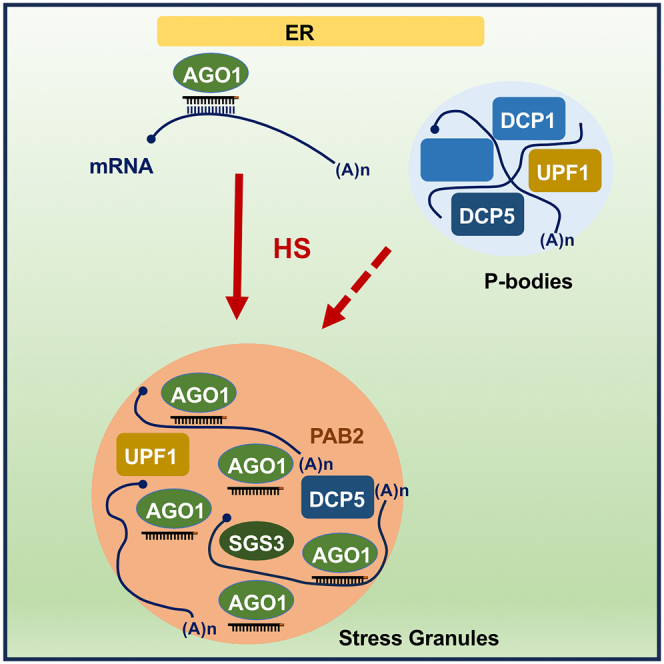

## Introduction

In eukaryotes, gene silencing is essential for development and plays important roles in response to stresses and for the epigenetic control of transposable elements.^1^ At the molecular level, RNA silencing involves the processing of double-stranded RNA by Dicer-like RNase III enzymes, into small RNAs (sRNAs) ranging from 21 to 30 nucleotides (nt) in length (21–24-nt in plants). Small RNA duplexes are then incorporated into a protein complex called RISC (RNA-induced silencing complex); RISC always contains a member of the highly conserved Argonaute (AGO) protein family.^2^^,^^3^ In mammals, Ago2 is the only catalytic AGO protein, which upon loading with sRNAs can engage in mRNA degradation and translational repression. Ago2 has been reported to localize to different membrane compartments, such as the Golgi body, the endoplasmic reticulum (ER), and multivesicular bodies (MVBs).^4^^,^^5^^,^^6^^,^^7^ While the functional relevance of some of these subcellular localizations still requires elaboration, it is clear that microRNA (miRNA)- and small interfering RNA (siRNA)-loaded Ago2 protein physically associates with the cytosolic side of the rough ER membrane to exert RNA silencing activity.^8^ The association of Ago2 to MVBs has also been linked to silencing,^6^^,^^7^ as this compartment seems required for RISC disassembly and may be involved in Ago2 secretion and/or lysosomal degradation. Besides these organelles, mammalian AGO proteins localize at specific non-membranous bodies, called P-bodies^9^^,^^10^^,^^11^^,^^12^^,^^13^^,^^14^^,^^15^ However, P-bodies are not required for RNA silencing.^16^^,^^17^ Under stress conditions Ago2 as well accumulates in other cytosolic bodies known as stress granules (SGs).^18^ Understanding the subcellular localization, trafficking, and function of AGO proteins under stress represents important current challenges.

In the model plant *Arabidopsis thaliana* (hereafter Arabidopsis), AGO1 plays a central role in both siRNA- and miRNA-directed silencing.^19^^,^^20^^,^^21^ AGO1 loaded with sRNA mediates cleavage of target transcripts^22^ and is required for translational repression of at least a fraction of them.^23^^,^^24^ In line with its essential role in RNA silencing, *ago1* mutants or depletion of the AGO1 protein severely compromises plant development and affects cell division.^25^^,^^26^^,^^27^ In addition, AGO1 is an important regulator of antiviral defense, as its mutation enhances susceptibility to different RNA viruses.^26^^,^^28^^,^^29^

At the subcellular level, it was recently reported that AGO1 carries both nuclear localization (NLS) and nuclear export signals (NESs). Unloaded cytosolic AGO1 has exposed NLS and hidden NES and is imported in the nucleus where it can be loaded with miRNAs, thus exposing its NES, which promotes its export to the cytosol in the form of miRNA-AGO1 complexes.^30^ In the nucleus, AGO1 has also been linked to other functions such as transcription and RNA-mediated DNA repair (reviewed by Bajczyk et al.^31^). Once in the cytosol, AGO1 appears in membrane-free (soluble) and membrane-bound forms, the latter being mainly the ER^24^^,^^32^^,^^33^^,^^34^ In fact, AGO1 association to the rough ER is the site of miRNA-mediated translational repression^24^ and biogenesis of phased siRNA on membrane-bound polysomes.^33^ Cytosolic AGO1 is also loaded with siRNAs produced in the cytosol, and the resulting complexes execute mRNA cleavage, which is exemplified in antiviral defense and post-transcriptional gene silencing (PTGS).^35^ Of note, PTGS also involves the production of secondary siRNAs via the action of the RNA DEPENDENT PROTEIN RNA POLYMERASE 6 (RDR6) and SUPPRESSOR OF GENE SILENCING 3 (SGS3), two components that reside in cytosolic foci called siRNA bodies.^36^ Whether Arabidopsis AGO1 associates with siRNA bodies or other cytoplasmic cellular compartments requires more investigation. Moreover, its subcellular distribution when plants encounter stress remains unclear.

Temperature is an important environmental factor that affects plant growth and development, and heat stress (HS) causes significant reduction in crop yield and quality.^37^^,^^38^ Previous work has shown that growing Arabidopsis plants permanently at 30°C inhibits PTGS mediated by the RDR6/SGS3 siRNA pathway.^39^ However, a recent report suggested that a short HS at 42°C stimulated the formation of siRNA bodies via SGS3 phase separation, leading to a massive production of endogenous siRNAs.^40^ In the present study we questioned how HS and its recovery affect AGO1 subcellular distribution and activity in RNA silencing. To avoid artifacts resulting from overexpression and/or transient expression assays, we performed most of our studies with Arabidopsis plants carrying genomic constructs of AGO1 and of other cellular compartment markers fused to fluorescent proteins and expressed under the control of their own regulatory elements. Using confocal laser scanning microscopy (CLSM) imaging of root meristematic cells, we show that upon HS at 37°C AGO1 dynamically associates in condensates corresponding to SGs. Besides SG proteins, the interactome of AGO1 upon HS also revealed P-body and RNA decay components. The recruitment of AGO1 in these condensates does not require SGS3; rather we show that the N-terminal Poly-Q domain of AGO1 has the ability to undergo phase separation, both *in vitro* and *in vivo*. Using transcriptomics, we observed that, while protein-coding genes are promptly responding to the HS and recovery condition, sRNAs are not impacted by short-term HS treatment. Additionally, we observed that miRNA accumulation and AGO1 loading or cleavage are not substantially affected by HS, suggesting that these AGO1 functions are not linked to AGO1 localization in SGs.

## Results

### AGO1 protein accumulates and dynamically associates with siRNA bodies during HS

**Figure 1:**
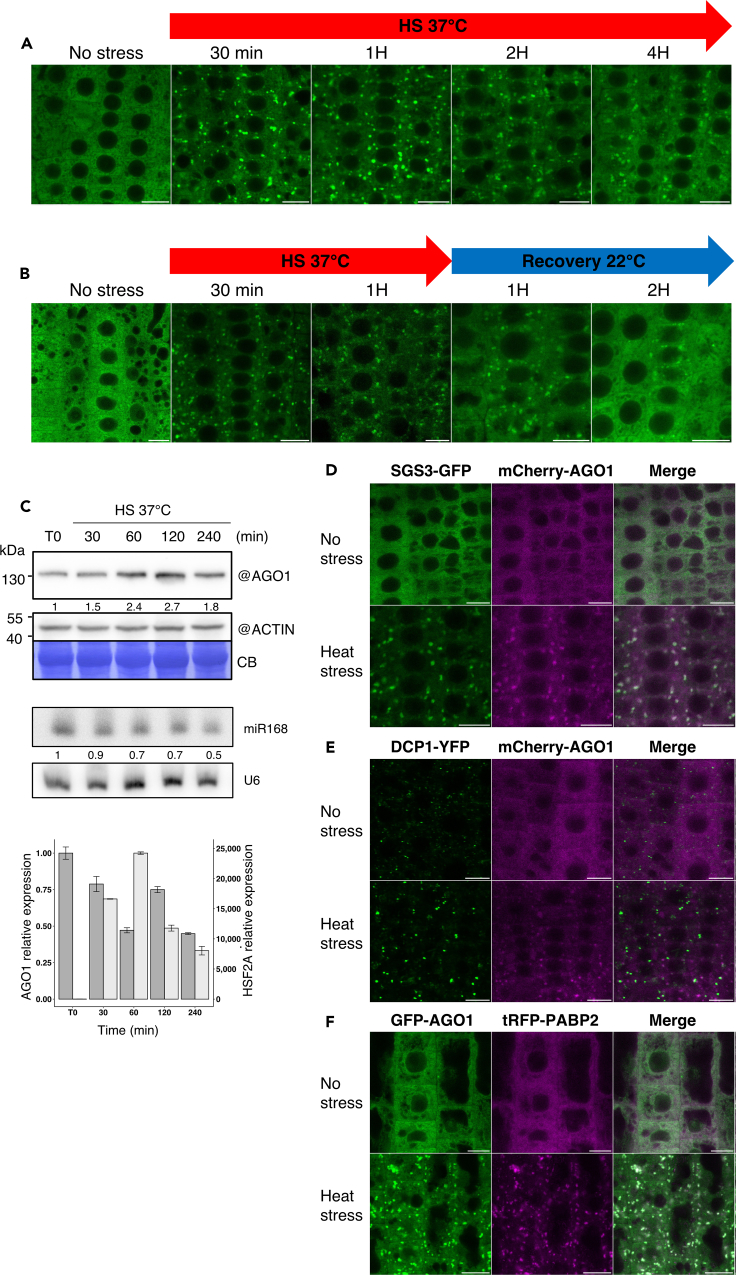
AGO1 protein accumulation and colocalization with the stress granules under HS (A and B) Dynamics of accumulation and disappearance of AGO1 foci during HS. CLSM imaging of 7-day-old Arabidopsis root tip cells of the pAGO1:GFP-AGO1 *ago1-27* transgenic line subjected to continuous HS (A) or 1 h HS at 37°C followed by 2 h recovery at 22°C (B). Bar = 10 μm. Objective 40×, oil immersion. See also Table S1. (C) AGO1 protein and RNA accumulation during HS. Kinetic analysis of 7-day-old Arabidopsis Col-0 seedlings grown on MS medium and subjected to HS. Upper panel: AGO1 protein was detected by western blot with a specific antibody. Coomassie blue (CB) staining and ACTIN were used as loading controls. AGO1 signal was quantified by ImageJ and normalized to the corresponding ACTIN signal. Numbers below the AGO1 panel are indicated as relative to non-stressed Col-0 (T0) set at 1.0. Middle panel: sRNA gel blot analysis of the steady-state accumulation of the indicated miRNA taken from the same material as above. U6 RNA level was used as a loading control. miRNAs signals were quantified by ImageJ and normalized to the corresponding U6 signal. Numbers below panels are indicated as relative to non-stressed Col-0 (T0) set at 1.0. Bottom panel: RT-qPCR analysis of AGO1 and HSFA2 transcript levels. RNA samples were extracted from the same material as above. Bars indicate the mean expression of three technical replicates +/− SD. (D) AGO1 colocalizes with SGS3 in foci under heat-stressed conditions. CLSM imaging of 5-day-old Arabidopsis pAGO1:mCherry-AGO1 x pRDR6:SGS3-GFP root tip cells before and after 37°C HS of 30 min. Experiments were performed in triplicate with three roots per replicate. Data are represented as mean +/− SME. Bar = 10 μm. Objective 40×, oil immersion. See also Table S1 and Video S1. Foci number after HS for SGS3-GFP = 842 ± 158; PCC = 0.77 ± 0.02. Foci number after HS for mCherry-AGO1 = 789 ± 111; PCC = 0.70 ± 0.04. (E) DCP1 does not colocalize with AGO1 under heat-stressed conditions. CLSM imaging of 5-day-old pDCP1:DCP1-YFP *dcp1-3* x pAGO1:mCherry-AGO1 Arabidopsis root tip cells before and after a 37°C HS of 30 min. Experiments were performed in triplicate with three roots per replicate. Data are represented as mean +/− SME. Bar = 10 μm. Objective 40×, oil immersion. See also Table S1. Foci number before HS for DCP1-YFP = 743 ± 115; PCC = 0.37 ± 0.03. Foci number after HS for DCP1-YFP = 690 ± 80; PCC = 0.31 ± 0.04. Foci number after HS for mCherry-AGO1 = 703 ± 70; PCC = 0.32 ± 0.02. (F) AGO1 colocalizes with PABP2 in foci under heat-stressed conditions. CLSM imaging of 5-day-old Arabidopsis pAGO1:GFP-AGO1 *ago1-27* x pPABP2:tRFP-PABP2 root tip cells before and after 37°C HS of 30 min. Experiment performed in triplicate with three roots per replicate. Data are represented as mean +/− SME. Bar = 10 μm. Objective 40×, oil immersion. See also Table S1. Foci number after HS for GFP-AGO1 = 1777 ± 142; PCC = 0.76 ± 0.04. Foci number after HS for tRFP-PABP2 = 2257 ± 194; PCC = 0.77 ± 0.03.

Under normal growth conditions and as previously reported, a functional GFP-AGO1 fusion protein is mainly localized in the cytoplasm of Arabidopsis root tip cells (Figure 1A).^30^^,^^34^^,^^41^ However, upon 30 min HS treatment at 37°C, AGO1 subcellular distribution substantially changed, with the appearance of GFP-AGO1 cytosolic foci that persisted for at least for 4 h during continuous HS (Figure 1A). Nonetheless, after HS, most of these foci disappeared during 2 h of temperature recovery at 22°C, and the diffuse AGO1 cytosolic pattern was restored (Figure 1B), indicating that HS-dependent AGO1 cellular relocalization is a dynamic process. We next investigated if HS affects AGO1 expression level and observed that the amount of AGO1 protein increased during HS in a transient manner (Figure 1C). The HS-dependent AGO1 protein accumulation did not correlate with an increase of AGO1 steady-state mRNA level but correlated with a lower accumulation of miR168, which guides AGO1 mRNA cleavage and translation repression^20^^,^^42^^,^^43^ (Figure 1C). Thus, heat-induced AGO1 protein accumulation occurs at the post-transcriptional level.

To identify intracellular organelles with which AGO1 may associate during HS, we crossed AGO1 reporter lines with Arabidopsis lines expressing different cytoplasmic compartment markers (Golgi, the *trans*-Golgi network/early endosome (TGN/EE), and MVB), allowing dual subcellular colocalization studies (see Table S1). Of particular interest are MVBs as AGO1 was recently found in extracellular vesicles (EVs), originating from this compartment.^44^ Moreover, MVBs also play important roles in trafficking proteins to the vacuoles^45^ and vacuolar degradation of AGO1, at least in a viral context, as has previously been reported in plants.^34^^,^^41^ Nonetheless, the fluorescence intensity between the AGO1 and MVB channels did not correlate in Arabidopsis root tip cells, confirmed by the low value of the Pearson coefficients (PPCs) (Figure S1A), indicating that GFP-AGO1 does not appear to colocalize with MVBs. Similarly, and despite a clear reorganization of these membranous organelles during HS, no colocalization of AGO1 with the TGN/EE and Golgi was observed (Figures S1B and S1C).

Next, we examined whether AGO1 associates with siRNA bodies upon HS. siRNA bodies are non-membranous cytoplasmic structures characterized by the presence of the proteins SGS3 and RDR6.^46^^,^^47^ AGO7, which participates in the production of *trans*-acting siRNA (ta-siRNA), is also found in siRNA bodies.^36^ Notably siRNA body formation was previously shown to be distinct from P-bodies and to dramatically increase under HS in leaves of *Nicotiana tabacum*.^36^^,^^48^ Thus, we crossed the pAGO1:mCherry-AGO1 line with the pRDR6:SGS3-GFP line (Table S1). Confocal imaging of Arabidopsis root cells indicated that under normal growth conditions the SGS3-GFP signal was mainly diffuse and that only a few siRNA bodies were present (Figure 1D). Once we transferred seedlings for 30 min at 37°C, we observed a massive accumulation of foci, in which mCherry-AGO1 and SGS3-GFP were colocalized, as supported by the high value of PPCs for both proteins (Figure 1D; Video S1).

Video S1. Confocal time lapse (6 frames of 10 s intervals), single slice video of SGS3-GFP and mCherry-AGO1 mutual dynamics in Arabidopsis root cells, related to Figure 1D and the STAR Methods section

### HS-induced AGO1 bodies colocalize with SGs

We next investigated the cellular interactions between AGO1-containing foci and two other membrane-less ribonucleoparticles (RNPs) composed of an association of mRNA and proteins: P-bodies and SGs. P-bodies are ubiquitously present in cells and contain RNA decapping enzymes and exonucleases and are known to play important roles in mRNA catabolism.^49^ Contrary to P-bodies, SGs are not present in non-stressed cells and form from mRNAs that become stalled in translation initiation during environmental stress such as HS.^49^^,^^50^ We first visualized P-bodies and SGs in our experimental conditions, by using an Arabidopsis transgenic line expressing DCP1-YFP and tRFP-PABP2, respectively, and confirmed that both entities do not colocalize under HS as previously reported (Figure S2A).^36^^,^^48^ Note that the mobility of P-bodies changes with stress; while they are very mobile under normal growth conditions, they become more static under HS (Videos S2 and S3) and are often found in close proximity to SGs, potentially allowing for crosstalk of the two entities.^47^^,^^48^

Video S2. Confocal time lapse (6 frames of 10 s intervals), single slice video of DCP1-YFP and tagRFP-PAB2 in non-stressed Arabidopsis root tip cells, related to Figure S2A and the STAR Methods section

Video S3. Confocal time lapse (6 frames of 10 s intervals), single slice video of DCP1-YFP and tagRFP-PAB2 in Arabidopsis root tip cells under HS, related to Figure S2A and the STAR Methods section

We next investigated the localization of mCherry-AGO1 with respect to the DCP1-YFP maker of P-bodies (Figure 1E). In the absence of stress, we did not observe a clear colocalization of these proteins, suggesting that AGO1, at least in Arabidopsis root tip cells, is not a major component of P-bodies. Moreover, during HS, the two proteins localized to distinct, not overlapping foci. On the other hand, GFP-AGO1 fully colocalized with the SG marker tRFP-PABP2 under HS (Figure 1F). This was also observed for SGS3-GFP (Figure S2B) and is in line with previous reports showing colocalization of siRNA bodies and SG markers after HS.^36^^,^^40^ Thus, the size of HS-induced AGO1-containing bodies and their low mobility and localization with PAB2 suggest that AGO1 is compartmentalized in SG condensates during HS.

**Figure 2:**
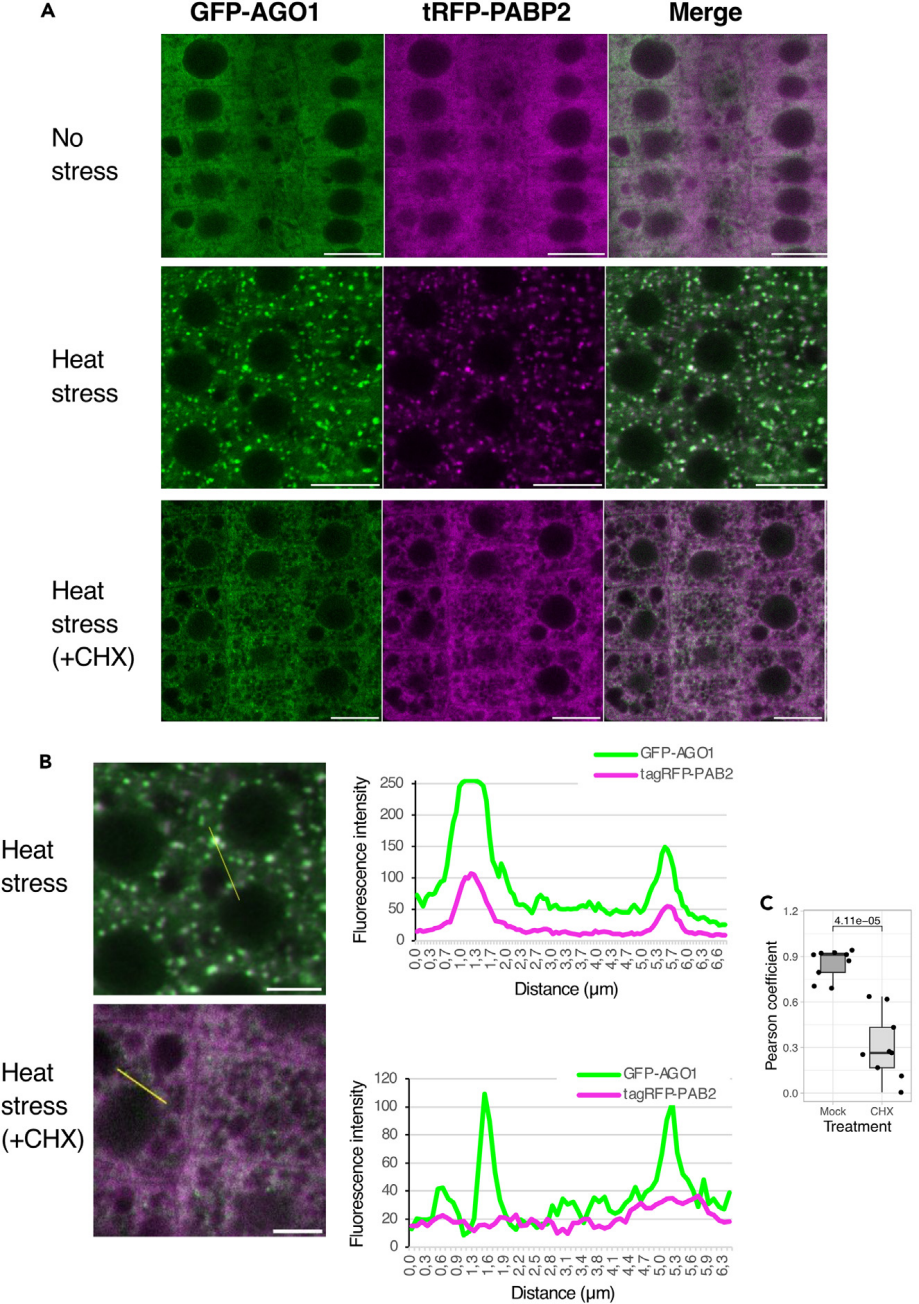
Cycloheximide inhibits stress granule formation, but allows the persistence of small AGO1 foci (A) CLSM imaging of 5-day-old Arabidopsis pAGO1:GFP-AGO1 *ago1-27* x pPABP2:tRFP-PABP2 root tip cells before HS (upper panels), after 37°C HS of 30 min (middle panels) and after 37°C HS of 30 min in the presence of 100μM CHX). Experiment performed in duplicate with three roots per replicate. Bar = 10 μm Objective 40×, oil immersion. Related to Figure 1F and see also Table S1 and Figure S3. (B) Signal intensity distribution of the total amount of pixels at the x axis shown in CHX-untreated and CHX-treated cells after 30 min of HS at 37°C shown in (A). Bar = 5 μm. (C) Quantification of the colocation observed between GFP-AGO1 fluorescence and tRFP-PABP2 after HS in non-treated and CHX-treated cells. Three independent roots were analyzed. For each of these roots, a selected area of a square of 15 μm side was defined in which 3 different measurements of PCC were performed. Data are represented as mean +/− SME for non CHX-treated plants (PC = 0.85 ± 0.03) and for CHX-treated plants (PC = 0.31 ± 0.07). p value shown has been obtained using a Wilcoxon rank-sum test.

Notably, SGs are sensitive to drugs inhibiting translation elongation such as cycloheximide (CHX), which prevents their formation in mammalian cells after drug treatment during stress.^51^^,^^52^ Thus, we investigated whether this is also the case for the HS-induced AGO1-containing bodies. Five-day-old seedlings expressing both GFP-AGO1 and tRFP-PABP2 were subjected to 37°C HS for 30 min in the absence or presence of 100 μM CHX (Figure 2). As expected, tRFP-PABP2 shows a diffuse cytosolic distribution in the presence of CHX, indicating the disassembly of SGs. Remarkably, HS-induced AGO1-containing bodies were still visible in the presence of CHX, though of smaller size (Figure 2). Similar results were also observed for an Arabidopsis line expressing both SGS3-GFP and tRFP-PABP2 (Figure S3), for which SGS3-decorated bodies were still visible under HS in the presence of CHX, in line with the recent report of Tan et al.^40^ Thus, while components of siRNA bodies and SG perfectly colocalize under HS, they also show some distinct features.

### Besides SG proteins some P-bodies and RNA decay components are also part of the HS-induced AGO1 interactome

**Figure 3:**
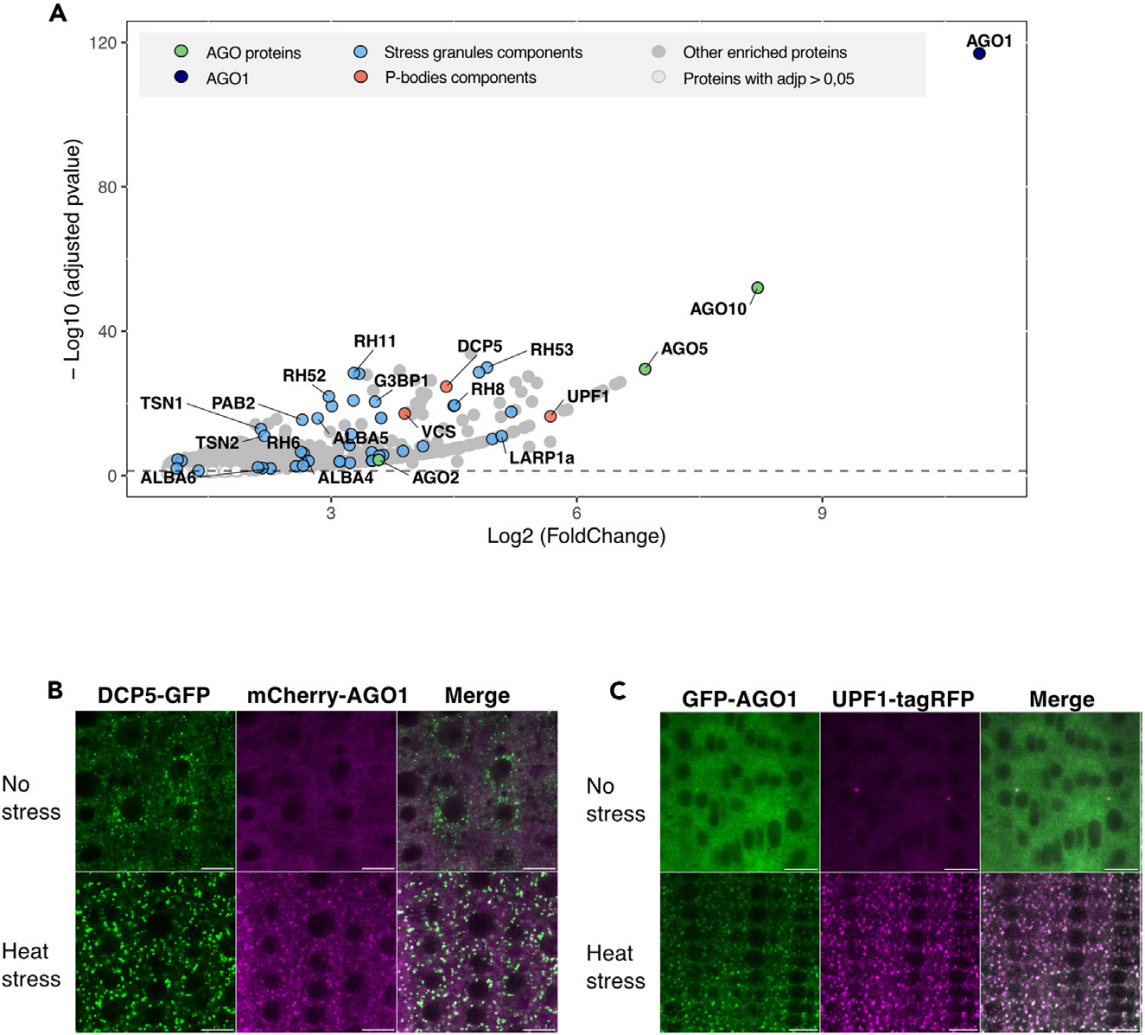
AGO1 interactome and its colocalization with P-body and RNA decay components under heat stress (A) AGO1 interactome revealed by immunoprecipitation and mass spectrometry. Seven-day-old Arabidopsis pAGO1:GFP-AGO1 *ago1-27* and Col-0 seedlings were subjected to 1 h HS at 37°C. We compared 12 samples (pAGO1:GFP-AGO1 *ago1-27*) from 4 independent biological replicates to 12 control samples (Col-0 seedlings subjected to 1 h HS at 37°C). Volcano plot shows the enrichment of proteins co-purified with the GFP-AGO1 bait compared with Col-0 controls. The y and x axes display log2 values from adjusted p values and fold changes, respectively. The horizontal dashed line indicates the threshold above which proteins are significantly enriched (adjusted p values <0.05). Only AGO1-enriched proteins (log2FC > 1) are shown. Three color-coded functional clusters are highlighted. Enriched proteins are dark gray, cytoplasmic RNA granules related proteins are highlighted in blue, AGO1 is in black and other AGO proteins are in green, selected P-bodies markers are in red. The source data are available in Table S2. See also Figure S4B. (B) DCP5 colocalize with AGO1 under heat-stressed conditions. CLSM imaging of 5-day-old Arabidopsis pDCP5:DCP5-GFP *dcp5-1* x pAGO1:mCherry-AGO1 root tip cells before and after 37°C HS of 30 min. Experiments were performed in triplicate with three roots per replicate. Data are represented as mean +/− SME. Bar = 10 μm. Objective 40×, oil immersion. See also Table S1. Foci number after HS for DCP5-GFP = 544 ± 60; PCC = 0.58 ± 0.03. Foci number after HS for mCherry-AGO1 = 429 ± 66; PCC = 0.53 ± 0.03. (C) AGO1 colocalizes with UPF1 in both normal and heat-stressed conditions. CLSM imaging of 5-day-old Arabidopsis pAGO1:GFP-AGO1(cs) *ago1-36* x pUPF1:UPF1-tagRFP *upf1-5* root tip cells before and after 37°C HS of 30 min. Experiments were performed in duplicate with three roots per replicate. Data are represented as mean +/− SME. Bar = 10 μm. Objective 40×, oil immersion. See also Table S1. Foci number before HS for UPF1-tagRFP = 69 ± 34; PCC = 0.67 ± 0.06. Foci number after HS for UPF1-tagRFP = 1344 ± 257; PCC = 0.76 ± 0.05. Foci number after HS for GFP-AGO1 = 1074 ± 269; PCC = 0.74 ± 0.05.

To identify the interaction network of AGO1 during HS, we immunoprecipitated GFP-AGO1 protein from Arabidopsis pAGO1:GFP-AGO1 *ago1-27* seedlings after 1 h at 37°C and performed mass spectrometry (MS) analysis. For this experiment, we used two different controls, either a p35S:GFP-3Flag transgenic line or wild-type (WT) Col-0 seedlings, which were both heat stressed in the same conditions as those of the pAGO1:GFP-AGO1 *ago1-27* line. Note that after 1 h HS at 37°C the line expressing GFP protein alone did not form the typical GFP-AGO1 foci, but the signal remains rather diffuse in both the nucleus and cytosol (Figure S4A). Proteins significantly enriched in the GFP-AGO1 immunoprecipitation (IP) were highlighted by a statistical analysis, calculating normalized fold changes and adjusted p values (Figure 3A, for the comparison to WT Col-0 seedlings; Figure S4B for the comparison to the p35S:GFP-3Flag line; see Table S2 for the whole dataset). In accordance with our microscopic observations, a large category of proteins predominantly enriched in the IP corresponded to SG components, including PAB2, RNA-binding protein 47A-C (RBP47A-C), RNA helicases (RH6/8/11/52/53), G3BP1, and TSN1/2, among others. In total, 72 proteins significantly enriched in our HS-induced AGO1-IP were shared with the reported Arabidopsis SG proteome.^53^

Surprisingly, we also identified some P-body and RNA decay components such as DCP5, VARICOSE, LARP1a, and UP-FRAMESHIFT1 (UPF1) in the AGO1 interactome. As AGO1 does not colocalize with DCP1 (Figure 1E), this raises the possibility that some, but not necessarily all components of P-bodies, might associate with HS-induced AGO1 condensates. To address this question, we generated Arabidopsis transgenic lines expressing mCherry-AGO1 in the pDCP5:DCP5-GFP *dcp5-1* background or GFP-AGO1 in pUPF1:UPF1-tagRFP *upf1-5* background (Table S1). Under non-stress conditions we did not observe colocalization between DCP5-GFP and mCherry-AGO1 (Figure 3B), but the proteins did colocalize during HS. To confirm that DCP5 colocalizes with SG, we also generated an Arabidopsis line expressing both DCP5-GFP and tRFP-PABP2. Under normal growing conditions only DCP5-GFP forms foci, likely corresponding to P-bodies, but, under HS, DCP5-GFP colocalized with tRFP-PABP2 in SGs (Figure S5A). For UPF1-tagRFP, we observed colocalization with GFP-AGO1 in few foci in the absence of HS, while under HS both proteins wholly colocalized (Figure 3C). Finally, we investigated the localization of SGS3-GFP with respect to UPF1-tagRFP and confirmed that during HS both proteins partially colocalize (Figure S5B), which is in accordance with previous observations showing partial colocalization of both proteins when transiently expressed in *Nicotiana benthamiana* (hereafter *N. benthamiana*) leaves.^54^ Overall, these data indicate that HS promotes the association of AGO1 with SG condensates together with siRNA bodies components and other proteins involved in RNA turnover, including the decapping-associated factor DCP5 and UPF1, a key component of nonsense-mediated mRNA decay (NMD).

### A short HS at 37°C has limited effect on sRNA accumulation and their loading into AGO1

**Figure 4:**
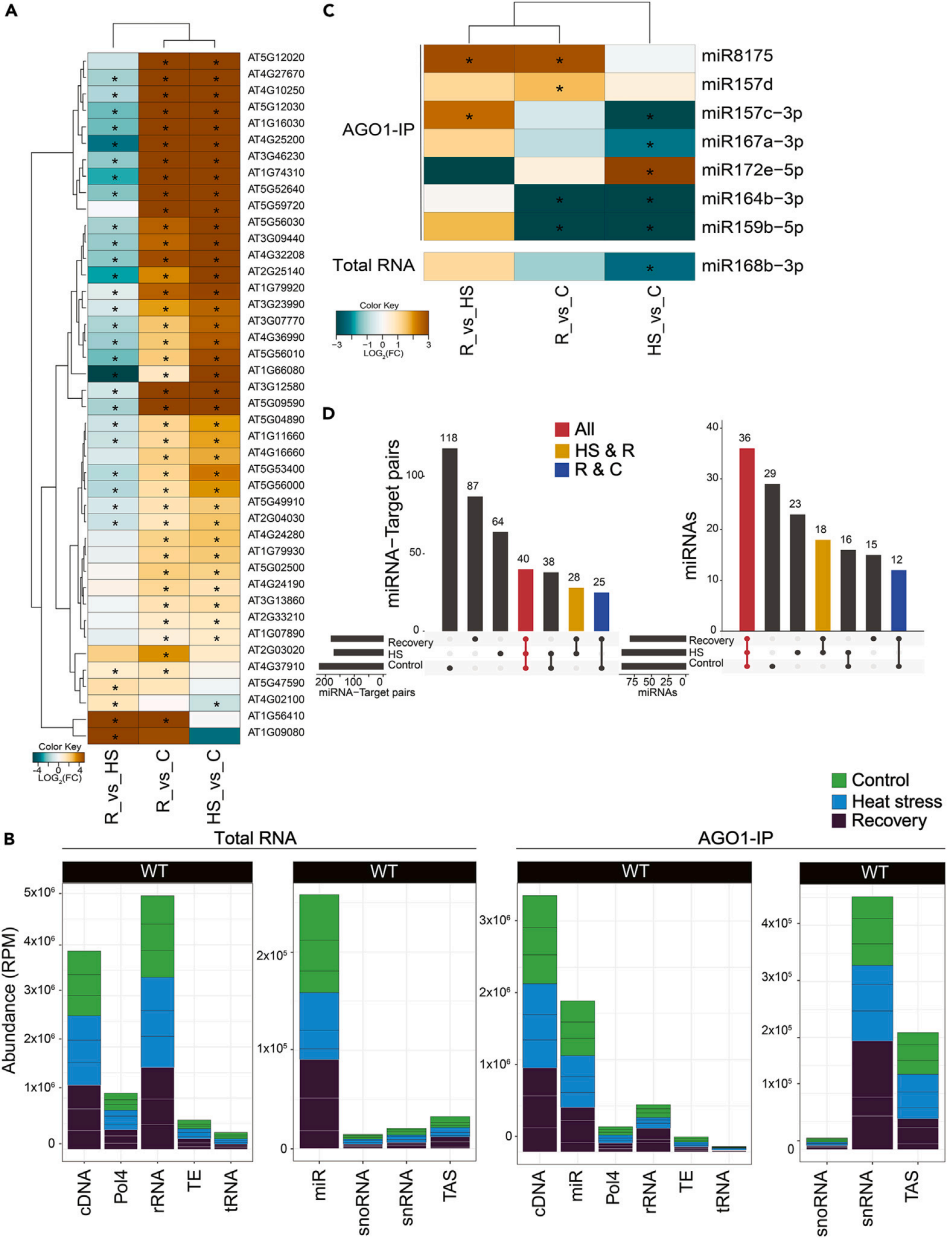
sRNA accumulation and loading into AGO1 during HS and recovery (A) Heat shock-related genes are differentially expressed in response to HS and recovery treatments. Heatmap of differentially expressed genes encoding heat shock-related proteins annotated in the TAIR10 genome. Brown indicates enrichment and teal indicates depletion in each of the three comparisons. C, Control; HS, Heat Stress; R, Recovery. See also Figure S6A. (B) sRNAs are not affected by HS. The sRNAs that mapped to the genome were categorized by origin annotated in TAIR10 and plotted by relative abundance in RPM. tRNA, Transfer RNAs; cDNA, complementary DNA; Pol IV, products dependent on RNA polymerase IV; snRNAs, small nuclear RNAs; snoRNA, small nucleolar RNA; TAS, *trans*-acting siRNA; rRNA, ribosomal RNAs. See also Figure S6B. (C) Eight miRNAs differentially accumulated in response to heat treatment and recovery. Heatmap of differentially expressed miRNAs in AGO1-IP (upper panel) and total RNA (lower panel). Brown indicates enrichment and teal indicates depletion in each of the three comparisons. C, Control; HS, Heat Stress; R, Recovery. (D) Upset plot representing the miRNA-target pairs (left panel) and miRNAs only (right panel) identified in nanoPARE sequencing for all three comparisons. For each upset plot, the bottom left shows the number of miRNA target pairs in each comparison as a horizontal histogram, the bottom right shows the intersection matrix and the upper right shows the size of each combination as a vertical histogram. The red color marks common pairs to all three comparisons, the yellow color marks common pairs between HS and recovery, and the blue lines mark common pairs between recovery and control. See also Figure S6D.

Next, we investigated how AGO1 loading and activity is affected by HS. To address this question, 7-day-old Col-0 seedlings grown at 22°C were subjected to HS at 37°C for 1 h, followed by 2 h recovery at 22°C. We performed sequencing analyses on coding RNAs, and total and AGO1-associated sRNAs. The transcriptomic analysis of the same RNA from WT Col-0 plants exposed to HS and recovery revealed that a total of 9,724 genes were differentially expressed in at least one of our three comparisons (Figure S6A; UpsetPlot). Out of this subset of genes, only 735 (or 7.5%, red) appear to be differentially expressed in all comparisons. The most transcriptional changes happened during the recovery phase since 6,626 (or ∼68%) genes appeared to be differentially expressed when comparing recovery to control, recovery to HS, or common to both comparisons (Figure S6A, blue). These genes were enriched in the “Response to Stress” GO category. As a reference subset, out of the 68 loci identified in TAIR (version 10) as heat shock proteins (HSPs) and heat shock factors (HSFs), 42 were differentially expressed in response to heat, including nuclear, mitochondrial, and chloroplastic HSPs (Figure 4A). Most of these heat shock-related genes were highly expressed when comparing HS versus control plants and remained at high levels when comparing recovery to control plants. However, when comparing recovery to HS plants, we observed their downregulation in accordance with the exit of the transcriptional HS response during recovery.

To assess the impact of HS on sRNAs, we generated sRNA libraries from total RNA and AGO1-IP on the same biological replicates used for the transcriptomic analysis. The sRNA libraries had the expected size distributions with peaks at 21–23- and 24-nt for total RNA and 20- and 21-nt for AGO1-IP, independent of the heat treatment (Figure S6B). sRNAs that mapped to the genome were categorized by origin and plotted by relative abundance (Figure 4B). Overall, no major differences in sRNA origin between treatments were observed. Moreover, there was no significant increase in any of the tasiRNA population (Figure S6C). We also analyzed the differential accumulation (DA) of each miRNA in each sample. For total RNA, only one miRNA (miR168b-3p) exhibited DA when comparing HS to control (Figure 4C). When analyzing the miRNAs loaded into AGO1, we found 7 miRNAs with DA in several samples. The majority of these miRNAs were downregulated during HS compared to control. We therefore conclude that neither the miRNA accumulation nor the loading in AGO1 changes dramatically in response to 1 h HS.

We next asked whether some miRNAs could target specific transcripts during HS and recovery. Thus, we generated nano-parallel analysis of RNA end (nanoPARE) libraries from the same material used for the sRNA sequencing (sRNA-seq) libraries. Considering only the miRNA target PARE present in two out of three biological replicates, we found that most of the miRNA target signatures detected are unique to each group (control, HS, and recovery) (Figure 4D, left panel). However, when comparing only miRNAs or targets that had a signature in nanoPARE, without considering their counterparts, we found that the majority of miRNAs were shared between all treatments (Figure 4D, right panel), while the majority of targets were unique to each treatment (Figure S6D). We conclude that there are few changes happening at the miRNA level and that the changes we observed might be happening at the transcript target level.

### AGO1 undergoes LLPS through its N-terminal domain

**Figure 5:**
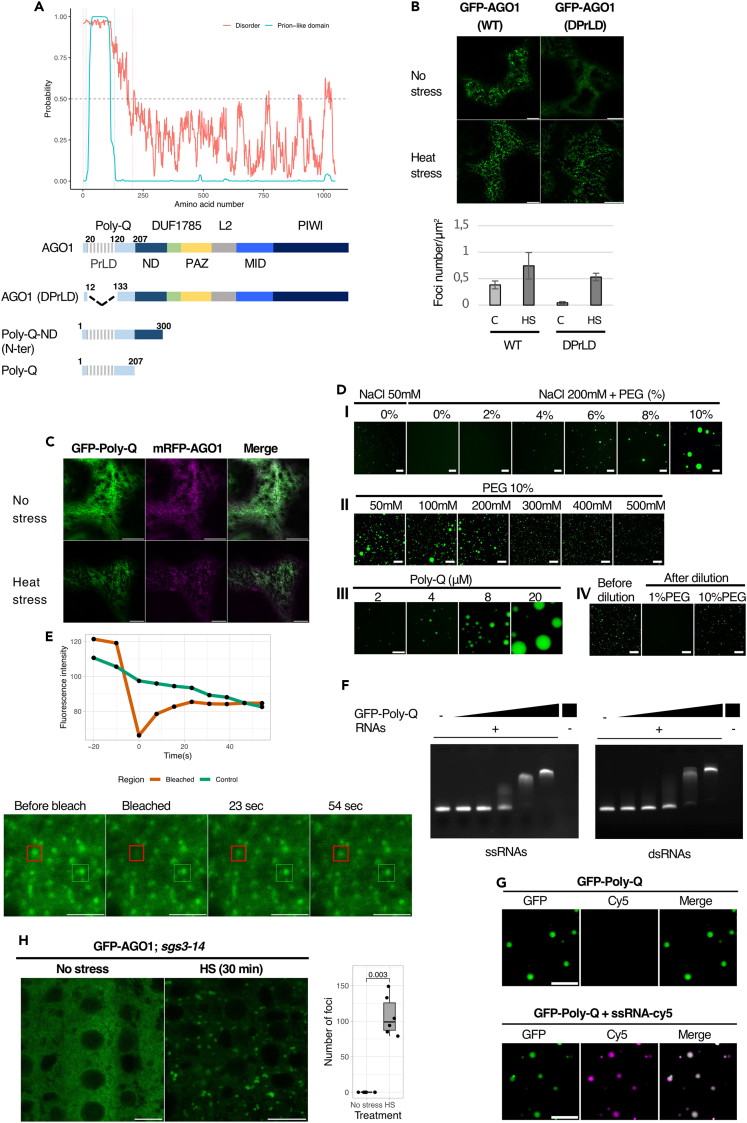
The N-terminal domain of AGO1 mediate HS-dependent LLPS (A) Schematic representation of the AGO1 deletion constructs used to assay LLPS in *N. benthamiana*. Each construct contains a GFP at the N-terminus and is under the control of the CaMV 35S promoter. The name of each AGO1 protein domain is indicated. The Poly-Q domain at the N-terminus is predicted to be both prion-like and intrinsically disordered. Prion-like domain and intrinsically disordered region were predicted using respectively http://plaac.wi.mit.edu/, https://iupred2a.elte.hu/. (B) The deletion of AGO1 PrLD does not abolish the efficiency for LLPS. Representative CLSM imaging of *N. benthamiana* leaf epidermal cells transiently expressing GFP-AGO1 (AGO1) and AGO1(ΔPrLD) proteins without or with HS (30 min at 37°C). Bar = 10 μm. Objective 40×, oil immersion. Bottom panel, quantification of GFP-labeled bodies for each construct per cytosolic area unit (foci number/μm^2^). Data are represented as mean ± SD. (C) The GFP-Poly-Q fusion protein colocalizes in the same foci as full-length mRFP-AGO1 under HS. CLSM imaging of *N. benthamiana* leaf epidermal cells transiently coexpressing p35S:GFP-Poly-Q and p35S:mRFP-AGO1 without or with HS (30 min at 37°C). Bar = 10 μm. Objective 40×, oil immersion. (D) AGO1 Poly-Q domain alone forms droplet-like structures *in vitro.* (I) *In vitro* analysis of droplet formation by recombinant GFP-Poly-Q protein at different PEG 8000 concentrations in 25 mM HEPES pH 7.5. Protein concentration = 4 μM. Bar = 20 μm. Objective 40×. (II) *In vitro* analysis of droplet formation by recombinant GFP-Poly-Q protein at different NaCl concentration in 25 mM HEPES pH 7.5, 10% PEG 8000. Protein concentration = 4 μM. Bar = 50 μm. Objective 20×. (III) *In vitro* analysis of droplet formation by recombinant GFP- Poly-Q protein at different protein concentrations in 25mM HEPES pH 7.5, 200 mM NaCl, 10% PEG 8000. Bar = 20 μm. Objective 40×. (IV) Reversion assay of droplets by 10-fold dilution of a concentrated (18 μM) GFP-Poly-Q in 25 mM HEPES pH 7.5, 200 mM NaCl, 10% PEG 8000 in solutions with or without PEG. Bar = 50μm. Objective 20×. See also Figure S8A. (E) FRAP assay of pAGO1:GFP-AGO1 *ago1-27* Arabidopsis root tip cells subjected to HS (30 min at 37°C). The fluorescence intensity during the time course of recovery after photobleaching and microscopy images are indicated. Time 0 indicates the time of the photobleaching pulse. Red and green scares indicate the photobleached and control condensates respectively. Bar = 10 μm. Objective 40×, oil immersion. Two additional independent experiments were conducted showing similar results (Figure S8B). (F) Agarose gel EMSA assay showing that GFP-Poly-Q domain binds ssRNA and dsRNA *in vitro*. RNAs are at 0.9 μM, GFP-Poly-Q protein ranges from 0,56 to 9 μM by 2-fold increment. (G) Colocalization of GFP-Poly-Q protein with Cy5-labeled-ssRNA upon droplet formation in 25mM HEPES pH7.5, 200mM NaCl, 10% PEG 8000. Protein and RNA are at 4 μM. Bar = 10 μm. Objective 40×, oil immersion. See also Figure S8D. (H) AGO1 does not rely on SGS3 to form condensate during HS. Left panel, representative CLSM imaging of 5-day-old Arabidopsis root tip cells of the pAGO1:GFP-AGO1 x *sgs3-14* transgenic line subjected to 30 min HS at 37°C. Bar = 10 μm. Objective 40×, oil immersion. Right panel, quantification of the relative number of condensates formed before and after HS. The number of foci was counted inside a comparable area of a square of 30 μm side of root cells from two biological replicates (with measurements of 3 independent roots each). Data are represented as mean ± SD. p value shown has been obtained using a Wilcoxon rank-sum test. See also Figure S9.

An intriguing question remains: how does AGO1 accumulate in condensates under HS? Recent work revealed that SGS3 has two prion-like domains (PrLDs) that mediate liquid-liquid phase separation (LLPS) in siRNA body formation.^40^^,^^55^ Interestingly, PrLDs have been predicted as protein motifs for Arabidopsis AGO1 and AGO2,^56^ but not characterized yet. For AGO1, the unique PrLD is located in the N-terminal part of the protein in a region called the Poly-Q domain due to its high glutamine content (Figure 5A). In fact, the Poly-Q domain encompasses an intrinsically disordered region (IDR), which is predicted with good probability to undergo LLPS. To investigate the importance of the AGO1 N-terminal region for its subcellular localization, we engineered an AGO1 construct lacking the PrLD (AGO1(ΔPrLD)). The native version of AGO1 and the mutated variant were expressed as GFP fusion proteins in *N. benthamiana* leaves subjected, or not, to 30 min of HS at 37°C. In contrast to the stable expression of GFP-AGO1 in Arabidopsis, a significant number of foci could already be observed with the AGO1 (WT) construct even in the absence of HS in *N. benthamiana* (Figure 5B). This is likely due to the stress resulting from the transient expression assay and/or the overexpression of AGO1. Even so, HS stimulated the formation of foci for both AGO1 constructs, indicating that this response does not depend on the sole PrLD.

Next, we investigated whether the AGO1 N-terminal region alone is sufficient to induce phase separation. We expressed in *N. benthamiana* as GFP fusion proteins either the entire N-terminal region (called Poly-Q-ND) or the Poly-Q domain alone (Figure 5A). For both constructs, efficient cytosolic foci formation was observed in an HS-dependent manner (Figure S7). Notably, these constructs displayed a filamentous pattern of the GFP signal that was attenuated under HS and was not observed with full-length GFP-AGO1. Importantly, co-expression of the GFP-Poly-Q together with mRFP-AGO1 confirmed that the HS-induced Poly-Q condensates correspond to those observed with full-length AGO1 (Figure 5C).

To investigate whether the ability to undergo LLPS depends on the properties of the Poly-Q region, we expressed it as an N-terminal GFP fusion in *Escherichia coli*, purified it to near homogeneity, and used *in vitro* phase transition assays. We found that the AGO1 Poly-Q domain, but not maltose-binding protein fused to GFP, was sufficient to rapidly drive GFP into droplet-like structures *in vitro* when incubated with the molecular crowding agent PEG 8000 (Figures 5D-I and S8A). At near-physiological salt concentration, droplet size increased with the PEG concentration from 4% to 10% and their formation was partly inhibited by NaCl concentration above 200 mM (Figure 5D-II). Lower salt concentration was, however, sufficient to initiate the formation of small droplets in absence of PEG (Figure 5D). Droplet size was also strongly influenced by protein concentration, ranging from 1 μm to 20 μm in diameter when AGO1 Poly-Q domain concentration was raised from 2 μM to 20 μM (Figure 5D-III). We next tested whether droplets formation was reversible by diluting 10-fold pre-formed GFP-Poly-Q droplets in buffers containing either 1% or 10% PEG. In assays where PEG concentration was reduced to 1%, the droplets mostly disappeared while they remained unchanged when PEG concentration was maintained at 10% (Figure 5D-IIIV), demonstrating that the process is reversible. To further assess the molecular dynamics and mobility of the AGO1 phase-separated liquid droplets in Arabidopsis, we subjected pAGO1:GFP-AGO1 *ago1-27* line to HS and performed fluorescence recovery after photobleaching (FRAP) assays. In line with the liquid-like nature of the AGO1 droplets, their FRAP was fast within a few seconds (Figures 5E and S8B). Similar results were obtained when FRAP assays were performed on *in vitro*-produced GFP-Poly-Q droplets (Figure S8C).

Since the Poly-Q domain contains several RGG/RG motifs^57^ recognized as RNA-binding motifs,^58^ we then investigated whether the presence of RNA had an influence on LLPS. Interestingly, recombinant GFP-Poly-Q domain of AGO1 protein could efficiently bind 112 nt long single-stranded and double-stranded RNA in electrophoretic mobility assays (EMSAs) (Figure 5F). Moreover, in LLPS assays, when Cy5-labeled RNA was co-incubated with Poly-Q domain in the presence of 10% PEG, RNA and proteins colocalized in the same droplets (Figures 5G and S8D). However, neither droplets size nor droplets abundance was affected suggesting that the interaction between the AGO1 Poly-Q domain and RNA does not facilitate LLPS transition but only enables co-recruitment of RNA in the droplets whose formation is largely mediated by the protein’s intrinsic properties.

### SGS3 is not required for AGO1 to undergo LLPS and localize in SGs

A previous report showed that RDR6 was not recruited to siRNA bodies in protoplasts of Arabidopsis *sgs3* mutants,^55^ suggesting that the formation of siRNA bodies requires SGS3. To investigate if the formation of condensates containing AGO1 and SGS3 together with SG components during HS also depends on SGS3, we introduced the pAGO1:GFP-AGO1 construct in the *sgs3-14* null mutant.^59^ Remarkably, after HS, the formation of AGO1 foci was not impaired in root tip cells, indicating that AGO1 does not rely on SGS3 for LLPS *in vivo* and for its localization to SGs (Figure 5H). This prompted us to reinvestigate the role of SGS3 for the recruitment of RDR6 during HS in root tip cells of stable transgenic Arabidopsis lines. For this, we analyzed transgenic lines constitutively expressing GFP-RDR6 or RDR6-GFP in both Col-0 and the *sgs3-1* null mutant.^60^ After 1 h HS at 37°C, RDR6 was able to form foci independent of the presence or absence of SGS3 (Figure S9), indicating that SGS3 is not essential for the recruitment of RNA silencing components to HS-induced SGs, at least in Arabidopsis root tip cells.

## Discussion

### During HS AGO1 localizes in SG condensates with siRNA body, P-body, and NMD components

In the present study, we investigated the subcellular distribution of AGO1 in plants subjected to HS. Under non-stressed growing conditions, we observed GFP-AGO1 localization mainly diffuses in the cytoplasm as previously reported.^30^^,^^34^^,^^41^ Moreover, we did not find an enrichment of the AGO1 signal in any of the endomembrane organelles we examined and that are known to be involved in vesicular trafficking and protein sorting, such as the TGN/EE and MVB. Our observation differs from a study that found that AGO1 is associated with EVs isolated from the apoplastic fluid of Arabidopsis leaves,^44^ suggesting AGO1 secretion through the MVB-exosome pathway. However, this contradiction may be explained by the different types of tissues used in the studies: leaf tissue in the former and root tip cells in our study.

We employed HS to investigate changes in AGO1 subcellular distribution as this stress triggers dramatic effects on gene expression and RNA metabolism.^61^^,^^62^ We focused our study on three non-membranous RNPs known to play important functions during HS (e.g., P-bodies, SGs, and siRNA bodies). In fact, HS blocks translation initiation by provoking 5′-ribosome pausing,^63^ and in Arabidopsis 25% of the paused RNAs undergo 5′-to-3′-mediated decay.^62^ This degradation is mediated by XRN4, an exoribonuclease that resides in P-bodies. However, paused RNA can also be stored in SGs, which prevent its degradation and allow translation in the recovery period after stress. This is particularly true for mRNA encoding ribosomal proteins (RPs) that are released from SG during recovery for the production of new ribosomes to restore the translation machinery.^64^ Thus, P-bodies and SGs seem to have distinct functions during HS (RNA degradation and RNA storage, respectively).

A third type of condensate, siRNA bodies, contains the essential actors of PTGS, which eliminates dangerous endogenous (transposons) and exogenous (virus) RNA.^65^^,^^66^ siRNA bodies, composed by the machinery of RDR6/SGS3, form small and discrete cytoplasmic foci which are eventually associated with membranes.^36^^,^^47^ While AGO7, required for the biogenesis of ta-siRNAs from the TAS3 precursor, has been shown to accumulate in siRNA bodies,^36^ the relationship of AGO1 with this type of compartment is less understood, despite the fact that AGO1 is a main player in the production of secondary siRNA, including ta-siRNA and pha-siRNA species. Notably, during stress, and particularly HS, it has been shown that siRNA body and SG components co-localize, but not with P-body components.^36^^,^^54^ Recently, a study reported that a driver of siRNA body formation is SGS3, and the purification of SGS3 condensates identified numerous RNA-binding proteins and components of the PTGS machinery.^40^

Here we showed that HS triggers a rapid redistribution of AGO1 from its diffuse cytosolic pattern to condensates that also contain siRNA body and SG components. Although microscopy indicated that AGO1 and SGS3 colocalized very well under HS, SGS3 was not found to be significantly enriched via AGO1-IP, and only a few SGS3 peptides were identified by MS. This may be explained by the low abundance of SGS3 protein and its reported potential degradation under HS.^67^ Consistent with this possibility, AGO1 could be co-purified with SGS3 when the latter was overexpressed.^40^ Moreover, both AGO1 (our work) and SGS3^40^ interactomes revealed a large number of components of SGs. For instance, we identified PAB8, RNA-binding protein 47A-C (RBP47A-C), RNA helicases (RH6/8/11/52/53), G3BP1, and TSN1/2, among others. Nevertheless, we found that although siRNA body and SG components colocalize after HS, these two structures also have distinct features. Indeed, CHX, a drug interfering with translational elongation, inhibits the formation of SGs but not of siRNA bodies. This raises questions about the assembly and dynamics of these entities and whether siRNA bodies remain distinct compartmentalized structures inside SGs.

Intriguing, and less expected, was the identification of some P-body and RNA decay components, such as DCP5 and UPF1, in the AGO1 interactome during HS. We confirmed the colocalization of both proteins with AGO1 during HS in stably transformed Arabidopsis plants. These results indicate that during HS P-bodies and SGs are not strictly separated entities but rather that some P-body components, and components of NMD, can be recruited to SGs. Interestingly, P-bodies and siRNA bodies were often found dynamically juxtaposed, potentially allowing for crosstalk of the two machineries.^47^^,^^48^ At present it is unclear if these proteins are active in the HS-induced condensates, or whether they affect PTGS activity and/or gene expression; however given the central role for UPF1 in post-transcriptional and translational gene regulation,^68^ these results deserve further investigation.

### LLPS and recruitment of AGO1 to SG condensates

A large body of previous work revealed that LLPS of proteins and RNAs are essential for the dynamic assembly of SGs.^69^^,^^70^^,^^71^ It is also well established that IDRs and PrLDs are driving forces for phase separation of many proteins.^72^^,^^73^ Thus, how condensates are assembled during HS in plants and how AGO1 is recruited to these entities are important questions to answer.

Interestingly, following HS we identified several proteins in the interactome of AGO1 which have the capacity to phase separate into SGs. Among them are ALBA proteins, which undergo LLPS to localize at SGs and were recently shown to confer thermotolerance in Arabidopsis.^74^ Nevertheless, loss of ALBA proteins does not affect the formation of SGs,^74^ indicating that these proteins are not essential for SG assembly. Other proteins identified in our interactome, such as the Tudor staphylococcal nuclease (TSN1 and TSN2) and RH8 and RH12, have been shown to contribute to the assembly of SG and P-body condensates.^75^^,^^76^ Whether these proteins influence the formation of AGO1-containing condensates during HS will require further investigation. Notably, recent work showed that SGS3 exhibits phase separation both *in vivo* and *in vitro* through its PrLDs, highlighting the importance of LLPS in siRNA body formation.^40^^,^^55^ These studies also suggested that SGS3 is essential for the recruitment of RDR6 to SGs; in WT Arabidopsis protoplasts, RDR6 was able to form siRNA body-like foci, while this was abolished in protoplasts of an *sgs3* mutant.^55^ Moreover, in a yeast heterologous system, it was shown that SGS3-GFP forms condensates and concentrates RDR6-mCherry, while RDR6-mCherry alone is unable to form such entities.^40^ However, in stable Arabidopsis transformants expressing RDR6 fused to GFP, the formation of siRNA body-like foci was still observed under HS, even in a *sgs3* null mutant, indicating that SGS3 is not always required. The discrepancy between our results and the previous reports might be explained by the different experimental settings.

Importantly, our work also revealed that AGO1 does not require SGS3 to efficiently form condensates during HS in Arabidopsis. Interestingly, the still poorly characterized N-terminal region of AGO1 contains a large IDR enriched in polar and charged amino acids, also called Poly-Q, which itself contains a PrLD. Nevertheless, the deletion of the PrLD did not abolish the capacity of AGO1 to form condensates during HS, at least in transient expression assays, indicating that other residues of the Poly-Q contribute to this capacity. The phase-separating behavior of GFP-AGO1 in heat-induced condensates in Arabidopsis cells was also supported by FRAP assays showing a dynamic recovery of the fluorescence after photobleaching. Moreover, the Poly-Q domain alone was sufficient to undergo phase separation both *in vitro* and *in planta*. Strikingly, we found that this domain is also able to bind single-stranded and double-stranded RNA, but the addition of RNA did not facilitate LLPS of the AGO1 Poly-Q domain. It was shown that for some RNA-binding proteins, RNA rather inhibits LLPS to prevent the aberrant formation of protein condensates.^77^ Whether the Poly-Q domain of AGO1 binds RNA under HS *in vivo*, and how this would affect AGO1 phase separation, will require further studies. Note that AGO1 may not be the only Arabidopsis AGO protein able to phase separate under HS given the presence of PrLDs in the N-terminal regions of AGO2 and AGO5^55^ and the identification of both proteins in the AGO1 interactome during HS. This raises the question of the physiological reasons for the compartmentation of AGO proteins, and for AGO1 in particular, in condensates during stress.

### Effects of a short HS treatment on AGO1 loading and activity

As indicated earlier, P-bodies and SGs have clearly defined function during HS (RNA degradation and RNA storage, respectively). Finding AGO1, but also SGS3 and SG components, in the same condensates raises the question of how PTGS is affected by HS and what may be its contribution to the HS response and/or recovery. A previous study reported that the efficiency of transgene PTGS-associated siRNAs and of endogenous ta-siRNAs is reduced when growing Arabidopsis plants permanently at 30°C due to the partial dysfunction of the siRNA body component SGS3.^39^ Another study reported that the level of Arabidopsis TAS1 ta-siRNAs is reduced and the level of TAS1 ta-siRNA targets increased after 1 h of HS at 37°C.^78^ Overexpression of TAS1 RNA reduced thermotolerance whereas downregulation of TAS1 RNA, or overexpression of TAS1 ta-siRNA targets, increased thermotolerance. Conversely, a recent study^40^ indicated that the liquid-like properties of SGS3 are essential for its function in siRNA production.^40^ The authors reported that 15 min of HS at 42°C or treatment with CHX, which both inhibit translation, increases the level of ta-siRNAs and causes the production of siRNAs from endogenous genes; similar outcomes are observed when P-body components are impaired suggesting that siRNA bodies are active during a short HS treatment.^40^ On the contrary, our results, from WT Col-0 seedlings subjected to 1 h HS at 37°C, revealed that although the transcriptome strongly responds to HS, changes at the sRNA level are quite minor. In particular, we did not observe a strong accumulation of 21/22 nt siRNAs from endogenous genes nor a significant increase in any of the ta-siRNA population. Furthermore, the relative abundance of most miRNA, or their loading in AGO1, was not significantly affected by the HS treatment. However, this does not exclude that some of the miRNAs could play a function during HS, as a number of unique miRNA target signatures were detected during HS and recovery. The discrepancy between our data and the recent report^40^ could be explained by the fact that the latter study used SGS3-overexpressing approaches, while we used WT Col-0 seedlings. It is also possible that the level of temperature and/or the length of the HS modulate siRNA production and PTGS activity. Indeed, when focusing only on ta-siRNA accumulation, a permanent growth at 30°C resulted in reduced accumulation,^39^ whereas 1 h at 37°C caused limited^78^ or no significant reduction (our work), contrasting a shorter but more intense HS (15 min at 42°C), which caused an increased accumulation.^40^ Overall, understanding the impact of increased temperatures on AGO1 and more globally on RNA silencing will be crucial to develop novel strategies to cope with climate change and global warming.

### Limitations of the study

In this manuscript we showed that upon HS, AGO1, the major effector of RNA silencing in plants, dynamically associates in condensates corresponding to SGs. We showed that the N-terminal Poly-Q domain of AGO1 undergoes LLPS both *in vitro* and *in vivo*. However, whether the deletion of the entire Poly-Q domain is sufficient to block phase separation of AGO1 will still need to be determined. Engineering Arabidopsis transgenic lines expressing AGO1 with the Poly-Q domain deleted would clarify this question. If such lines are viable and would indeed exhibit impaired AGO1 localization in condensates during HS, these plants would also be very valuable to further explore the physiological significance of AGO1 phase separation during stress. We have shown that AGO1 localization in condensates barely affects its loading by miRNAs and cleavage activity, suggesting that this localization protects the protein from stress. It will be interesting to determine whether other abiotic or even biotic stresses have similar effects on AGO1. Given the importance of PTGS in plant development, stress responses, and antiviral defense, it will also be essential to determine to which extent it is affected by HS at various temperatures (30°C, 37°C, 42°C) and over different exposure times. Another aspect that remains unclear is whether AGO1 localization in condensates requires post-translational modifications. Finally, it is possible that AGO1 phase separation into SG, even with little change in the composition of its loaded sRNA, could still have important regulatory functions. Notably, recent work in metazoans revealed that phase separation of Ago2 could promote the recruitment of deadenylation components of the CCR4-NOT complex^79^ or allows the recruitment of an E3 ubiquitin ligase to catalyze nascent-peptide ubiquitylation as part of a protein quality control process.^80^ Whether Arabidopsis AGO1 condensates are involved in such or other regulatory processes will need further investigation.

## STAR★Methods

### Key resources table

**Table:** 

REAGENT or RESOURCE	SOURCE	IDENTIFIER
**Antibodies**		

Rabbit polyclonal anti-AGO1	Agrisera	Cat# AS09 527, RRID:AB_2224930
Mouse monoclonal anti-GFP (JL8)	Clontech (Takara)	Cat# 632381, RRID:AB_2313808
Rabbit polyclonal anti-ACTIN	Agrisera	Cat# AS13 2640, RRID:AB_2722610
Peroxidase-conjugated goat anti-rabbit IgG	Thermo Fisher Scientific	Cat# G-21234, RRID:AB_2536530
Peroxidase-conjugated goat anti-mouse IgG	Thermo Fisher Scientific	Cat# G-21040, RRID:AB_2536527

**Bacterial and virus strains**		

*Escherichia coli*	Invitrogen	Top10
*Escherichia coli*	Novogene	BL21
*Agrobacterium tumefaciens*		C58C1
*Agrobacterium tumefaciens*		GV3101 Pmp90

**Chemicals, peptides, and recombinant proteins**		

Cycloheximide (use at 100 μM)	Sigma Aldrich	01810
MS medium	Duchefa	MO255
Clarity Western ECL substrate	Biorad	1705061
RQ1 RNase-free DNase	Promega	M6101
deoxynucleotide triphosphate (dNTP)	Promega	U1205
Protease inhibitor: complete-EDTA free	Roche	04693132001
Igepal CA-630	Sigma Aldrich	I8896
Formaldehyde	Fisher Scientific	28906
Glycogen	Fisher Scientific	R0561
Syringic acid (also called acetosyringone)	Sigma Aldrich	S6881
μ Columns	Miltenyi Biotech	130-042-701
Criterion TGX 4-15% gradient precast gels	Biorad	5671084
Nupage 4–12% gradient precast gels gels	Fisher Scientific	10472322
T4 Polynucleotide Kinase	Thermo Fisher	EK0031
Sequencing Grade Modified Trypsin	Promega	V5117
PerfectHyb Plus hybridization buffer	Sigma Aldrich	H7033
Hybond-NX Amersham	GE-Healthcare	RPN203T
*N*-(3-Dimethylaminopropyl)-*N*′-ethylcarbodiimide hydrochloride	Sigma Aldrich	E7750
PureProteome Protein A Magnetic Bead System	Sigma Aldrich	LSKMAGA
Cy5-allyl-UTP	Jena Bioscience	NU-821-CY5

**Critical commercial assays**		

RealSeq v2 kit	Barberán-Soler et al.^81^	RealSeq®-AC
μMACS GFP Isolation Kit	Miltenyi Biotech	130-091-125
Superscript III Reverse transcriptase kit	Invitrogen	Cat N° 18080093
Takyon™ No ROX SYBR 2X MasterMix blue dTTP kit	Eurogentec	UF-NSMT-B0701

**Deposited data**		

Mass spectrometry proteomics data deposited to the ProteomeXchange Consortium via the PRIDE (Perez-Riverol et al., 2019) partner repository.	PRIDE	PXD048594 and https://doi.org/10.6019/PXD048594Table S2
Data for RNA seq sRNA seq	NCBI’s Gene Expression Omnibus Edgar et al.^82^	GEO Series accession number GSE239837 Table S5
Data for sRNA seq	NCBI’s Gene Expression Omnibus Edgar et al.^82^	GEO Series accession number GSE239837 Table S6
Data for NanoPARE	NCBI’s Gene Expression Omnibus Edgar et al.^82^	GEO Series accession number GSE239837 Table S7

**Experimental models: Organisms/strains**		

Nicothiana benthamiana		N/A
Arabidopsis thaliana ecotype Colombia		N/A
*Arabidopsis* : pAGO1:GFP-AGO1(cs) *ago1-27*	Derrien et al.^41^	N/A
*Arabidopsis* : pAGO1:mCherry-AGO1	Bologna et al.^30^	N/A
*Arabidopsis* : pAGO1:GFP-AGO1*ago1-27*	Bologna et al.^30^	N/A
*Arabidopsis* : pRDR6:SGS3-GFP	This manuscript	N/A
*Arabidopsis* : p35S :RDR6-GFP *sgs3-1*	Moreno et al.^54^	N/A
*Arabidopsis* : pDCP1:DCP1-YFP *dcp1-3*	Chicois et al.^83^	N/A
*Arabidopsis* : pPABP2:tRFP-PABP2	Merret et al.^84^	N/A
*Arabidopsis* : pUB10:DCP5g-GFP *dcp5-1*	Chicois et al.^83^	N/A
*Arabidopsis* : pUPF1:UPF1g-tagRFP *upf1-5*	Chicois et al.^83^	N/A
*Arabidopsis* : pUB10:mCherry-RabG3f (R5)	Geldner et al.^85^	N/A
*Arabidopsis* : pUB10:mCherry-Rha1/RabF2a (R7)	Geldner et al.^85^	N/A
*Arabidopsis* : pUB10:mCherry-VTI12 (R13)	Geldner et al.^85^	N/A
*Arabidopsis* : pUB10:mCherry-SYP22 (R22)	Geldner et al.^85^	N/A
*Arabidopsis* : *sgs3-14*	Peragine et al.^59^	SALK_00139
*Arabidopsis* : p35S:GFP-3FLAG	This manuscript	N/A

**Oligonucleotides**		

RT-QPCR primers	Table S3, this paper	N/A
Primers for genotyping	Table S3, this paper	N/A
Probe sequence	Table S3, this paper	N/A
Primers for cloning	Table S3, this paper	N/A

**Software and algorithms**		

ImageJ version 1.53a	Schneider et al.^86^	https://imagej.nih.gov/
Lightcycler 480 software, Release 1.5.0 SP3	Roche	Cat. No. 04994884001
RStudio v.1.2.1335	RStudio Team	https://github.com/rstudio/rstudio
R version 3.6.3	R Core Team	https://www.R-project.org/
R packages used for Volcano Plot	ggplot2 (v3.4.1)	https://ggplot2.tidyverse.org
R packages used for Volcano Plot	ggrepel (v0.9.2)	https://github.com/slowkow/ggrepel
R packages used for Volcano Plot	dplyr(v1.1.1)	https://github.com/tidyverse/dplyr
R packages used for barplot and boxplot	gridExtra(v2.3)	https://CRAN.R-project.org/package=gridExtra
R packages used for barplot and boxplot	cowplot(v1.1.1)	https://wilkelab.org/cowplot/
Small RNA libraries trimming	Cutadapt(v2.9) Martin^87^	https://github.com/marcelm/cutadapt/
Nanopare analysis	Martin^87^	http://www.github.com/Gregor-Mendel-Institute/nanoPARE
RNAseq libraries analysis	HISAT2 Kim et al.^88^	https://github.com/DaehwanKimLab/hisat2
RNAseq libraries analysis	StringTie pipeline Pertea et al.^89^	https://ccb.jhu.edu/software/stringtie/
RNAseq libraries analysis	DESeq2 Love et al.^90^	https://github.com/thelovelab/DESeq2

### Resource availability

#### Lead contact

Requests for resources and reagents should be directed to and will be fulfilled by the lead contact, Pascal Genschik (pascal.genschik@ibmp-cnrs.unistra.fr).

#### Materials availability

Transgenic plant seeds generated in this study are available from the lead contact on request.

#### Data and code availability

• Availability of proteomics and RNA-seq data through online repertories: The mass spectrometry proteomics data have been deposited to the ProteomeXchange Consortium via the PRIDE^91^ partner repository with the dataset identifier PXD048594 and https://doi.org/10.6019/PXD048594. RNA deep sequencing data have been deposited in NCBI’s Gene Expression Omnibus^82^ and are accessible through GEO Series accession number GSE239837.
• This paper does not report original code.
• Any additional information reported in this paper is available from the lead contact upon request.

### Experimental model and study participant details

*Arabidopsis thaliana* ecotype Colombia as well as *Nicotiana benthamiana* (for transient expression assays), were used in this study. The Arabidopsis mutants, *ago1-27*^26^ and the *sgs3-14* null mutant^59^ were used. Transgenic Arabidopsis lines used in this work which were previously published^30^^,^^41^^,^^85^^,^^83^^,^^84^ are reported in Table S1. This Table also provides information on the subcellular localization of the fluorescent proteins expressed. Note that the pAGO1:GFP-AGO1(cs) *ago1-36* line^41^ was only used for the cross with pUPF1:UPF1-tagRFP *upf1-5*, while all other crosses were performed with the pAGO1:GFP-AGO1 *ago1-27* line.^30^ The p35S:RDR6 line^54^ was crossed with the sgs3-1 mutant.^60^ Two transgenic Arabidopsis lines, pRDR6:SGS3-GFP and p35S:GFP-3Flag, were generated for this study. The cloning procedure of the two constructs used to establish them is described below. The list of primers used for genotyping and cloning are presented in Table S3.

### Method details

#### Plasmid constructions

The list of primers used for cloning is presented in Table S3.

pENTRY(221)-Poly-Q: To obtain this construct, the N-terminal sequence of AGO1 (CDS) corresponding to the Poly-Q domain of AGO1 (amino acid 1 to 207) was amplified from the vector pENTRY(ZEO)-AGO1 (CDS) with oligo primers containing AttB1 and AttB2 recombination sites (primers called AGO1 Poly-Q-fwd and AGO1 Poly-Q-rev, see Table S3) and the sequence was mobilized into the pDONR221 vector by BP Gateway recombination (Invitrogen).

pENTRY-R2-Poly-Q-ND-L3: To obtain this construct, the N-terminal sequence of AGO1 corresponding to the Poly-Q-ND domain of AGO1 (amino acid 1 to 300) was amplified from the vector pENTRY-R2-AGO1 genomic-L3 with oligo primers containing AttB3 and AttB2R recombination sites (primers called attB2R_AGO1g_F and attB3NterAGO1-300_rev, see Table S3) and the sequence was mobilized into the pDONOR-P2RP3 vector by BP Gateway recombination (Invitrogen).

The pENTRY(ZEO)-AGO1(DPrLD) vector has been obtained by mutagenesis using pENTRY(ZEO)-AGO1(CDS) as a template by the company GenScript. In this construct a sequence of 119 amino acids (amino acid 13 to 132) encompassing the PrLD domain has been deleted from WT AGO1 (CDS).

The p35S:GFP-Poly-Q construct was obtained by Gateway LR recombination (Invitrogen) using pENTRY(221)-Poly-Q and the binary vector pB7WGF2 (^92^; https://gatewayvectors.vib.be/collection). This construct expresses a GFP-Poly-Q fusion protein under the regulation of the 35S promoter.

The p35S:GFP-Poly-Q-ND construct was obtained by assembling the 35S promoter (pENTRY-R4-35S-L1), the GFP (pENTRY(221)-GFP) and the Poly-Q-ND sequence of AGO1(pENTRY-R2-Poly-Q-ND-L3) into the binary vector pB7m34GW (https://gatewayvectors.vib.be/collection) by the three-way LR Gateway reaction (Invitrogen). This construct expresses the GFP-Poly-Q-ND of AGO1 fusion under the control of the 35S promoter.

The p35S:GFP-AGO1(DPrLD) construct was obtained by Gateway LR recombination (Invitrogen) using pENTRY(ZEO)-AGO1(DPrLD) and the binary vector pB7WGF2 (^92^; https://gatewayvectors.vib.be/collection). This construct expresses the GFP-AGO1(DPrLD) fusion protein placed under the regulation of the 35S promoter.

The p35S:mRFP-AGO1 construct was obtained by Gateway LR recombination (Invitrogen) using pENTRY(ZEO)-AGO1(CDS)^93^ and the binary vector pB7WGR2^92^; https://gatewayvectors.vib.be/collection. This construct expresses the mRFP-AGO1(CDS) fusion protein under the regulation of the 35S promoter.

The pRDR6:SGS3-GFP construct was obtained by a two-step process. First pRDR6-pGWB4 was obtained by cloning RDR6 promoter in pGWB4^94^ digested by HindIII after amplification by the primers pairs proSGS2F-hIII / proSGS2R-HIII (see Table S3). In parallel the cDNA of *SGS3* was cloned in the GATEWAY™ compatible vector pDONR207 (Invitrogen) using the following primers: attB2SGS3f/ attB2SGS3R (see Table S3). Finally, SGS3 was transferred to the binary vectors pRDR6-pGWB4 by Gateway LR recombination (Invitrogen) to make the pRDR6:SGS3-GFP.

The p35S:GFP-3Flag construct was obtained by a multi-step cloning process. First, we modified the multiple cloning site of Pily vector^95^ by insertion of annealed oligos 612PacAscF and 612PacAscR (see Table S3) in HindIII and SmaI digested Pily vector to create Pily-PA-7Ha. The 3xFlag sequence was obtained by oligonucleotides annealing using primers 612FlagF and 612FlagR (see Table S3), and cloned into Pily-PA-7Ha after XbaI and EcoRI digestion to obtain Pily-PA-3Flag. We amplified eGFP sequence by PCR using primer eGFPNheIF and eGFPNheIR (see Table S3) then digested with NheI and cloned in XbaI digested Pily-PA-3Flag to obtain Pily-PA-GFP-3FLAG. Finally, Pily-PA-GFP-3Flag and pART27^96^ were digested by NotI in order to transfer GFP-3Flag sequence in the binary vectors pART27 to obtain the p35S:GFP-3Flag.

#### Plant growth conditions and treatments

For *in vitro* culture conditions, Arabidopsis seeds were surface-sterilized using ethanol and plated on MS agar (MES-buffered MS salts medium [Duchefa, Murashige & Skoog medium inc. vitamins/MES- MO255)], 1% sucrose, and 0.8% agar, pH 5.7). The seeds were then stratified for 2 days at 4°C in the dark and then transferred in 16h light/8h dark (20,5/17°C,70% humidity) growth chamber, under fluorescent light (Osram Biolux 58W/965). The plants that grew on soil were under a 16h light/8 h dark diurnal regime.

For root cell microscopy, seeds were grown on MS-agar plates that were positioned vertically in the growth chamber.

For HS treatment, 7-day old seedlings were mounted on microscopy slides, that were transferred into an incubator set at 37°C for 30 minutes.

For cycloheximide treatments, Arabidopsis seedlings were grown for 5 days on MS-agar plates then transferred into liquid 1/2MS medium, 0,5g/L sucrose supplemented with either 100μM of cycloheximide (CHX) or methanol (0,02%) (Mock) for 30 minutes. These seedlings were then mounted on microscopy slides and put in an incubator heated at 37°C for 30 minutes.

For western blot, qRT-PCR, Northern blot and IP-MS experiments, Col-0 and pAGO1:GFP-AGO1 *ago1-27* lines were grown for 7 days on MS-agar plates then exposed to 37°C for 1 hour. In order to get the most homogeneous heat treatment, the MS-agar plates were wrapped into a plastic bag and submerged for 1 hour into a water bath set at 37°C. The plates were transferred into a second water bath set at 22°C for 2hours when recovery experiments were performed.

#### Transient expression in N. benthamiana leaves

Agrobacterium cells (GV3101 Pmp90 or C58C1) harboring the constructs of interest were grown overnight at 28°C in 10mL LB medium supplemented with antibiotics, resuspended in 10mM MgCl_2_ supplemented with 200mM acetosyringone at an OD of 0,3 per construct (unless otherwise specified), and incubated for 1 hour at room temperature before being pressure infiltrated into leaves of 4 week-old plants. All agro-infiltration assays were conducted in the presence of P19. Plants were maintained in growth chambers under 16 hours light and 8 hours dark photoperiod with a constant temperature of 22°C. Sampling of leaf disc was performed using a mechanical sampler and observations of 3 leaf discs, each coming from a different leaf and plant, were performed 48 hours after agro-infiltration. Agroinfiltrated leaves were heat stressed by incubation in a previously heated at 37°C 10mM MgCl_2_ buffer for 1 hour at 37°C in darkness.

#### Confocal microscopy analysis, quantifications and statistical analysis

Tobacco leaf (abaxial epidermal cells) and Arabidopsis root cells were imaged by CLSM using a Leica SP8 microscope. Fluorescence Recovery After Photobleaching (FRAP) assays were performed using a Zeiss LSM780 microscope. Arabidopsis seedling roots were positioned between a microscopy slide and a cover slip containing 0.5MS 0.5g/L sucrose liquid media, while hypocotyls and cotyledons were left exposed. Usual excitation/detection-range parameters for GFP and mCherry, mRFP or tagRFP were 488 nm/500–550 nm and 561 nm/600–650 nm, respectively and emissions were collected using the system’s hybrid (Hyd) and photomultiplicator detectors. When GFP and mRFP/mCherry/tagRFP were simultaneously imaged in transient expression assays, excitation/detection-range parameters were 488 nm/500–550 nm and 561 nm/600–650 nm, respectively. Sequential scanning was employed at all times. Images were processed and analyzed using ImageJ.^86^

Quantification of GFP or RFP labeled bodies was done semi-automatically using the ImageJ macro termed “2CH_bar” described previously.^34^ In each sample, density of foci per cell was calculated by first measuring the surface size covered by GFP signal (in μm2) and dividing the number of foci by the GFP surface size (number of foci/μm2). The 2CH_bar macro also allowed to determine the degree of correlation between green (GFP/YFP) and red (mCherry/tagRFP/mRFP) tagged bodies. For all samples and in each channel, data (XY coordinates and relative green/red fluorescence intensity) were gathered on each tagged body recognized by the macro which indicates also the total number of foci spotted. Then, global Pearson Coefficient Calculation was performed for each channel using relative green/red fluorescence intensity obtained for each spotted dot. Data are presented as mean ± SEM.

#### Protein analysis and western blotting

Proteins were extracted in pre-heated (95°C) 2X Laemmli sample buffer (62 mM Tris HCl pH 6.8, 3% SDS, 40% glycerol, 0,1% bromophenol blue, DTT 100mM, quantified using amido-black staining^97^ and 20μg of total proteins were separated by SDS-PAGE, either on 7–12% Tris-glycine gels or gradient NuPAGE 4–12% Bis-Tris Protein Gels (Thermo Fischer) or gradient Criterion TGX gel (4–15%) (BioRad). The list of antibodies are reported in the key resources table. For all western blots, immuno-luminescence was detected using the ECL Clarity (BioRad) and imaged using Fusion FX (Vilber).

#### Protein immuno-precipitation assays

For each GFP-IP, 1g of seedlings was ground in liquid nitrogen for 10 minutes in 3ml of ice-cold lysis buffer (50mM Tris-HCl pH7,5, 50mM NaCl, 0,25% IGEPAL CA-630, 2mM MgCl2, 5mM DTT, protease inhibitors (Complete™–EDTA free, Roche). The supernatant was cleared by 2 centrifugation steps of 15min then 5min at 10,000g at 4°C. The cleared supernatants were divided in 3 affinity purifications, incubated with magnetic microbeads coupled to GFP antibodies (μMACS™ GFP Isolation Kit, Miltenyi Biotec) and complexes were eluted in 100μl of pre-warmed elution buffer. IP experiments were performed in either three or four independent biological replicates. Each biological replicate was divided into three affinity purification replicates. In parallel control IPs were carried out with GFP antibodies in Col-0 and the p35S:GFP-3Flag line.

For immunoprecipitation of endogenous AGO1, 1g of frozen tissues was ground to a fine powder with a mortar and pestle, resuspended in 3 volumes of crude extract buffer (50mM Tris, pH 7,5, 150mM NaCl, 10% glycerol, 5mM MgCl2, 0,2% IGEPAL, 5mM DTT, and 1x Complete™–EDTA free (Roche)) and incubated for 20 min at 8 rpm in the cold room. Insoluble material was removed by centrifugation (two centrifugation steps at 10,000g at 4°C, first for 15 min then for 5 min). Identical amounts of crude extracts were incubated with prebound @AGO1 (5μg of @ AGO1 from Agrisera) PureProteome Protein A magnetic beads (30μL; Millipore) for 2 hr at 7 rpm at 4°C. Immune complexes were washed four times in the crude extract buffer, and purified sRNA was eluted from the beads in Tri-Reagent (Sigma-Aldrich) following the manufacturer’s instructions. Extracted RNA was precipitated in 2 volumes of isopropanol + 40 μg glycogen overnight at −20°C. RNA was resuspended in sterile water.

#### Mass spectrometry analysis, data processing and availability

Eluted proteins were digested with sequencing-grade trypsin (Promega, Fitchburg, MA, USA). Each sample was further analyzed by nanoLC-MS/MS on a QExactive+ mass spectrometer coupled to an EASY-nanoLC-1000 (Thermo-Fisher Scientific, USA) as described previously.^98^ Data were searched against the TAIRv10 fasta protein sequences from *Arabidopsis thaliana* with a decoy strategy (27.282 forward protein sequences). Peptides and proteins were identified with Mascot algorithm (version 2.8, Matrix Science, London, UK) and data were further imported into Proline v2.0 software (http://proline.profiproteomics.fr/). Proteins were validated on Mascot pretty rank equal to 1, and 1% FDR on both peptide spectrum matches (PSM score) and protein sets (Protein Set score). The total number of MS/MS fragmentation spectra was used to quantify each protein from at least six independent biological and affinity replicates. After a DEseq2 normalization of the data matrix, the spectral count values were submitted to a negative-binomial test using an edgeR GLM regression through R (R v3.2.5). For each identified protein, an adjusted pvalue (adjp) corrected by Benjamini–Hochberg was calculated, as well as a protein fold-change (FC). The results are presented in a Volcano plot using protein log2 fold changes and their corresponding adjusted p-value (-log10adjp) to highlight enriched and depleted proteins (see also Table S2).

#### Total RNA extraction

Total RNAs were extracted from 7-d-old seedlings grown on MS agar plates using TRI Reagent (Sigma) following the manufacturer’s instructions. RNAs were precipitated O/N at -20°C in 2 volumes of isopropanol + 40 μg glycogen. Quality and concentration of purified RNAs were assessed using Nanodrop and Qubit fluorometer.

#### RT-qPCR

For RT-qPCR, 2 μg of total RNA treated with DNase RQ1 (PROMEGA) was reverse transcribed with Superscript IV (Invitrogen) using a mix of random hexamers and oligo d(T). Each quantitative PCR reaction was performed in three technical replicates with Takyon™ SYBR® 2X qPCR Mastermix Blue (Eurogentec) in 384-wells plates with a total volume of 10 uL using Light Cycler 480 apparatus (Roche). mRNA abundance was compared to two reference genes EXP (AT4G26410) and TIP41 (AT4G34270) (Table S3). mRNA relative abundance was calculated using the ΔΔCt method. All oligonucleotide sequences used for RT-qPCR are listed in Table S3.

#### Low molecular weight northern blot

Northern blot analyses of low molecular weight RNAs were performed with 15 μg of total RNA resuspended in a final concentration of 50% (v/v) formamide-5mM EDTA-0.05% (w/v) bromophenol blue-0.05% (w/v) xylene cyanol, heated at 95°C for 2 min, and separated by electrophoresis on 15% polyacrylamide gels (19:1 acrylamide:bisacrylamide), 8 M Urea, 0.5× TBE gel. RNA was then transferred on Hybond-NX (Amersham) membrane and crosslinked with EDC for 1h30 at 60°C. DNA oligonucleotides complementary to sRNA and U6 were end-labeled with [γ-32P]ATP using T4 PNK (ThermoFisher). Membranes were incubated at 42°C with PerfectHyb Plus hybridization buffer (Sigma-Aldrich) for 30 min, hybridization was performed overnight in PerfectHyb Plus containing the radiolabelled probe at 42°C. The sequences of the Northen blot probes are described in Table S3). Membranes were washed three times in 1× SSC-1% SDS before exposure to a Fujifilm imaging plate. Signals were visualized with an Amersham Typhoon IP Biomolecular Imager (GE Healthcare Life Sciences).

#### ssRNA and dsRNA substrate preparation

A 112 nt long product from the GFP sequence was PCR amplified from plasmid pB7WGF2 with two oligos pairs (A349/A347 and A346/A348) using Phusion polymerase (ThermoFisher). The PCR products were purified on gel, and used as template in two separate T7 polymerase *in vitro* transcription reactions to generate sense and antisense transcripts. For Cy5 labelled ss and dsRNA, the *in vitro* transcription reaction of the sense transcript was spiked with 1mM Cy5-allyl-UTP (Jena Bioscience). RNA transcripts were purified from the reaction with the RNA clean-up Kit (Macherey-Nagel), quantified with a Qubit fluorometer (ThermoFisher) and verified on agarose gel. Double stranded RNA was obtained by equimolar mixing and annealing of both reverse-complementary transcripts.

#### Recombinant Poly-Q of AGO1 and MBP domain expression and purification

The Arabidopsis AGO1 DNA sequence encoding the prion-like motifs and intrinsically disordered regions of the N-terminal domain (amino acid 2 to 207) was amplified by PCR from a previously validated cDNA with two pairs of oligos (AGO int R, AGO1int F, AGO1-pQ B4 F and AGO1-pQ B4 R, see Table S3) in order to delete an internal *BsaI* site and generate a sequence compatible with GoldenGate cloning. The amplified products were digested, re-ligated within the *BsmBI* sites of the pUPD2^99^ vector before being further assembled into the *BsaI* sites of a customized GoldenGate-compatible pET expression plasmid to generate the final GFP-AGO1pQ domain-6xHis construct under the regulation of a T7 promoter. The GFP-MBP control plasmid was assembled similarly with the *E.coli* Maltose-binding-protein sequence replacing Poly-Q sequence to generate GFP-MBP-6xHis.

GFP-Poly-Q-6xHis and GFP-MBP-6xHis expressions were realized in BL21 *E. coli* strain in auto-inducing medium^100^ at 20°C for 18h. Cells were collected by centrifugation, resuspended in 25mM HEPES pH7.4, 500mM NaCl, 5mM beta-mercaptoethanol, and lyzed by three passages on a microfluidizer LM-20 (Microfluidics) at 1200 bars at 4°C. The lysates were clarified by centrifugation at 18000g for 30min at 4°C and the supernatants loaded on a NiNTA affinity column (HisTrap FF crude, Cytiva) equilibrated in 25mM HEPES pH7.4, 500mM NaCl, 25mM Imidazole and eluted in the same buffer with 250mM imidazole. The proteins were further purified through a size exclusion chromatography Superdex 200 16/60 column (Cytiva) equilibrated in 250mM HEPES pH7.4, 500mM NaCl. Fractions corresponding to monomeric proteins (apparent MW of 130 kDa for GFP-Poly-Q protein and 100kDa for GFP-MBP protein) were collected and concentrated by ultrafiltration, flash frozen in liquid nitrogen and stored at -80°C until use. Quality of proteins were assessed on 12% Tris-glycine SDS-PAGE gel, stained with Coomassie blue. Quantification was done by UV absorbance at 280nm on a Nanodrop 2000 (Thermo Scientific).

#### *In vitro* phase separation assay

For *in vitro* liquid droplet formation, purified protein was brought to the desired concentration through dilution into the reaction buffer containing 25mM HEPES pH 7.5, 200mM NaCl and 10% PEG 8000 (unless specified otherwise). 5ul droplets were placed on a glass slide and sealed in a small chamber, to prevent evaporation. After a 15min incubation period, imaging was performed under an LSM-700 Zeiss confocal microscope using 20X or 40X objectives. For co-localization experiment, 10x concentrated Cy5-labelled single stranded and double-stranded RNAs were added to the reaction together with the protein and the PEG containing dilution buffer, mixed thoroughly and incubated for 15min before observation. GFP and Cy5 were excited at 488 and 633nm, respectively, and detected at 490-612nm and 638-759nm, respectively.

#### FRAP assays

FRAP analyses were performed on an LSM-780 Zeiss using a 40X objective on samples prepared as specified above. For *in vivo* FRAP assays, photobleaching was achieved with the laser set at 488nm and 405nm, at 40% and 17,6% intensity, respectively, and 140 iterations).

For *in vitro* FRAP assays photobleaching was achieved with the laser set at 488nm, 40% intensity and 120 iterations).

#### EMSA

Recombinant GFP-Poly-Q of AGOI protein and *in vitro* transcribed RNAs were produced as described above, except that RNA was not fluorescently labeled. 9pmol of RNA was mixed with the specified amount of protein (5,6-20pmol by 2-fold increase) in 10ul volume reaction containing HEPES 25mM pH7.5, NaCl 50mM. After incubation at 25°C for 15min, the reaction was resolved on a 1% agarose gel, 0,5X Tris Borate EDTA buffer containing 1ug/ml ethidium bromide, at 100V, during 45min at RT. Gel documentation was done in a Fusion FX system, with the excitation at 365nm and the emission signal recorded at 595nm.

#### Libraries preparation and high-throughput sequencing

Total RNA samples were extracted from 1-week-old Col-0 seedlings (Col-0 seedling +/- 1hour treatment at 37°C (HS) and after an additional 2H period back at 22°C (Recovery)) grown on MS-agar plates using Tri-Reagent according to the manufacturer’s instruction. For AGO1-loaded sRNA samples, AGO1-IPs were performed as described above from 1g of 1-week-old Arabidopsis seedlings grown on MS-agar plates and following similar HS and recovery treatments. AGO1-loaded sRNAs were then extracted by adding Tri-Reagent directly on the magnetic beads and extraction of RNA was performed according to the manufacturer’s instructions. RNAs were precipitated O/N at -20°C in 2 volumes of isopropanol + 40 μg glycogen as described above.

Small RNA seq libraries were generated using RealSeq v2 kit,^81^ following manufacturer’s instructions, with 100ng as initial input and 15 cycles PCR amplification. nanoPARE and RNAseq libraries were generated following the protocol published by.^101^ All libraries were sequenced using Illumina NextSeq technology at the Delaware Biotechnology Institute (DE, USA). For small RNA libraries, we trimmed adapters and low-quality reads using Cutadapt v2.9 software^87^ and retaining only reads between 18- and 34-nt long. Reads were then mapped to the Arabidopsis genome version 10 (available at www.arabidopsis.org/download/) and its corresponding TAIR10 blastsets for all the features, using Bowtie2.^102^ NanoPARE libraries were analyzed using the pipeline provided by the authors (http://www.github.com/Gregor-Mendel-Institute/nanoPARE). RNAseq libraries were analyzed unsing the HISAT2 and StringTie pipeline.^89^^,^^88^ Differential accumulation and expression were done using DESeq2^90^ and all plots were generated using ggplot2^103^ packages in R environment.

Additional information for the sequencing experiments is presented in Tables S4, S5, S6, and S7. Table S4: Summary table for all sequencing experiments; Table S5: RNAseq summary table; Table S6: Small RNA count summary; Table S7: Nano-PARE summary.

### Quantification and statistical analysis

Barplot in Figure 1C, Volcano plot in Figure 3A and the line chart in Figure 4A have been generated with R version 3.6.3, running under macOS Sierra 10.12.6. Packages used for the volcano plot were: ggplot2 (v3.4.1), dplyr(v1.1.1) and ggrepel (v0.9.2). Additional packages used for barplot and boxplot were gridExtra(v2.3) and cowplot(v1.1.1).

## Supplemental information

Document S1. Figures S1–S9 and Tables S1, S3, and S4

Table S2. Proteins identified in control and coIP samples, related to Figures 3 and S4B

Table S5. RNA-seq summary table, related to Figures 4 and S6

Table S6. Small RNA count summary, related to Figures 4 and S6

Table S7. NanoPARE summary, related to Figures 4 and S6
