# Supplementary material for: Heat stress promotes Arabidopsis AGO1 phase separation and association with stress granule components
Source: iScience. 2024 Feb 6;27(3):109151. doi: 10.1016/j.isci.2024.109151 (PMC10879784; doi:10.1016/j.isci.2024.109151)
Supplement: Document S1. Figures S1–S9 and Tables S1, S3, and S4 [file mmc1.pdf]

iScience, Volume *27*

Supplemental information

Heat stress promotes Arabidopsis AGO1 phase separation and association with stress granule components

Aleksandar Blagojevic, Patricia Baldrich, Marlene Schiaffini, Esther Lechner, Nicolas Baumberger, Philippe Hammann, Taline Elmayan, Damien Garcia, Hervé Vaucheret, Blake C. Meyers, and Pascal Genschik

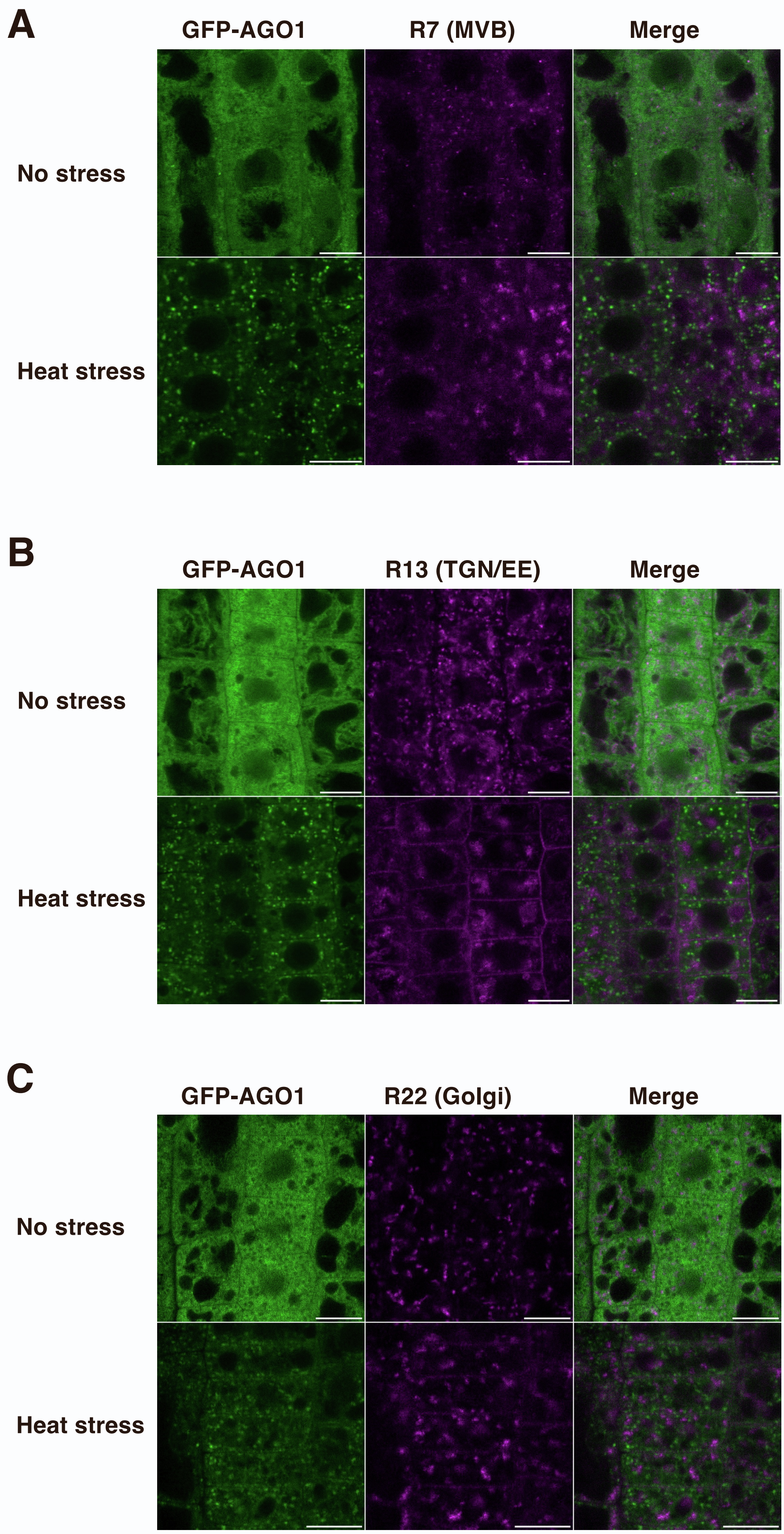
A
GFP-AGO1
R7 (MVB)
Merge
No stress
Heat stress
B
GFP-AGO1
R13 (TGN/EE)
Merge
No stress
Heat stress
C
GFP-AGO1
R22 (Golgi)
Merge
No stress
Heat stress

Figure S1

Fig. S1 Dual subcellular localization of AGO1 with different cytoplasmic compartment markers.

(A) AGO1 does not colocalize with the MVB marker R7 in root tip cells. See also Table S1 and the STAR Methods section. CLSM on 5-day old Arabidopsis pAGO1:GFP-AGO1 *ago1-27* x pUB10:mCherry-Rha1/RabF2a (R7) root tip cells before and after 37°C HS of 30 minutes. Experiments were performed in triplicate with three roots per replicate. Data are represented as mean +/- SME. Bar = 10 µm.

Foci number before HS for R7 = 1451 ± 109; PCC= 0.34 ± 0.04

Foci number after HS for R7 = 1049 ± 104; PCC= 0.29 ± 0.05

Foci number after HS for GFP-AGO1 = 1525 ± 168; PCC= 0.19 ± 0.03

(B) AGO1 does not colocalize with the TGN/EE marker R13 in root tip cells. See also Table S1 and the STAR Methods section. CLSM on 5 day old Arabidopsis pAGO1:GFP-AGO1 *ago1-27* x pUB10:mCherry-VTI12 (R13) root tip cells. Before and after 37°C HS of 30 minutes. Experiments were performed in triplicate with three roots per replicate. Data are represented as mean +/- SME. Bar = 10 µm.

Foci number before HS for R13 = 1311 ± 169; PCC= 0.12 ± 0.06

Foci number after HS for R13 = 553 ± 125; PCC= 0.07 ± 0.03

Foci number after HS for GFP-AGO1 = 1370 ± 155; PCC= 0.04 ± 0.03

(C) AGO1 does not colocalize with the Golgi marker R22 in root tip cells. See also Table S1 and the STAR Methods section. CLSM on 5 day old Arabidopsis pAGO1:GFP-AGO1 *ago1-27* x pUB10:mCherry-SYP22 (R22) root tip cells before and after 37°C HS of 30 minutes. Experiments were performed in triplicate with three roots per replicate. Data are represented as mean +/- SME. Bar = 10 µm.

Foci number before HS for R22 = 1355 ± 169; PCC= 0.47 ± 0.04

Foci number after HS for R22 = 662 ± 120; PCC= 0.28 ± 0.06

Foci number after HS for GFP-AGO1 = 1157 ± 250; PCC= 0.27 ± 0.05

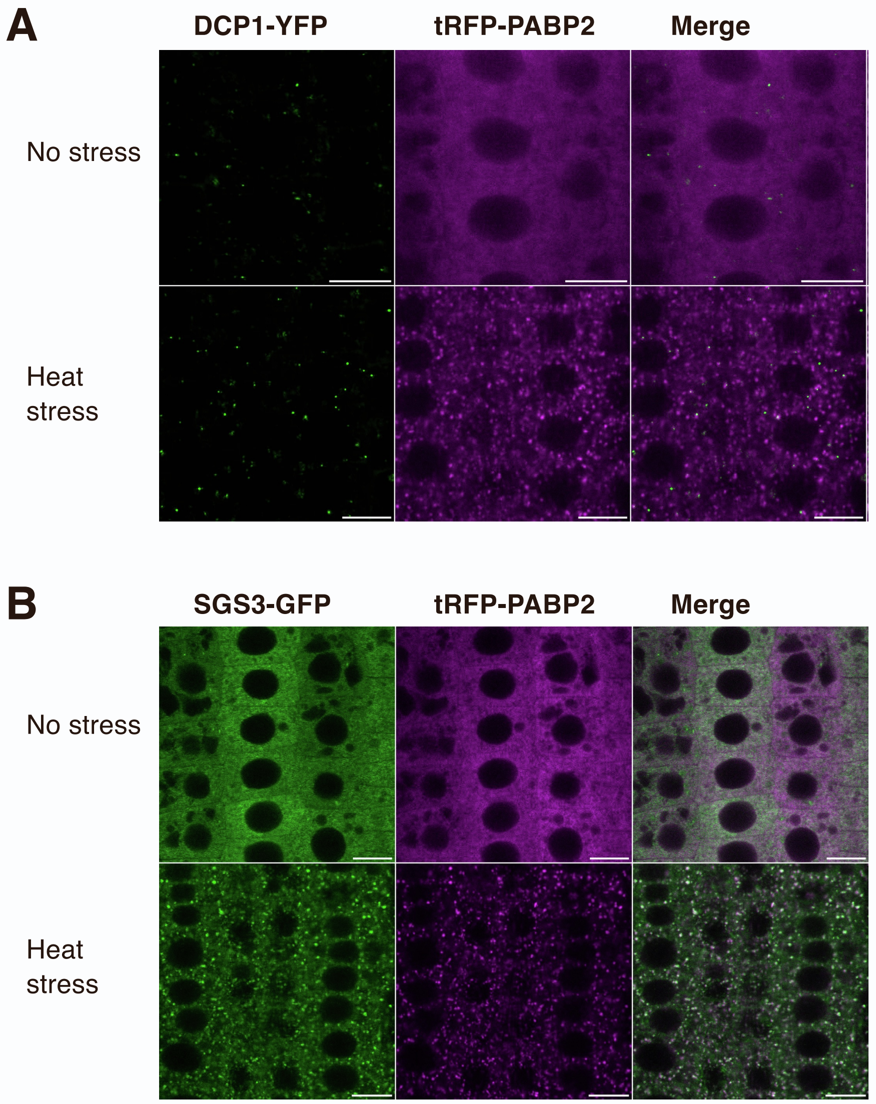

Fig. S2 The stress granule marker PAB2 colocalizes in foci with SGS3, but not DCP1 under HS.

(A) CLSM imaging of 5 day old Arabidopsis pDCP1:DCP1-YFP *dcp1-3* x pPABP2:tRFP-PABP2 root tip cells before and after 37°C HS of 30 minutes. See also Table S1 and the STAR Methods section. Experiments were performed in triplicate with three roots per replicate. Data are represented as mean +/- SME. Bar = 10 μm. Objective 40X, oil immersion.

Foci number before HS for DCP1-YFP = 1300 ± 153; PCC= 0.15 ± 0.03

Foci number after HS for DCP1-YFP = 478 ± 50; PCC= 0.06 ± 0.05

Foci number after HS for tRFP-PABP2 = 1666 ± 132; PCC= 0.34 ± 0.04

(B) CLSM imaging of 5 day old Arabidopsis pRDR6:SGS3-GFP x pPABP2:tRFP-PABP2 root tip cells before and after 37°C HS of 30 minutes. See also Table S1 and related to Figure 1F. Experiments were performed in triplicate with three roots per replicate. Data are represented as mean +/- SME. Bar = 10 μm.

Foci number after HS for SGS3-GFP = 1385 ± 156; PCC= 0.84 ± 0.01

Foci number after HS for tagRFP-PABP2 = 2152 ± 227; PCC= 0.85 ± 0.01

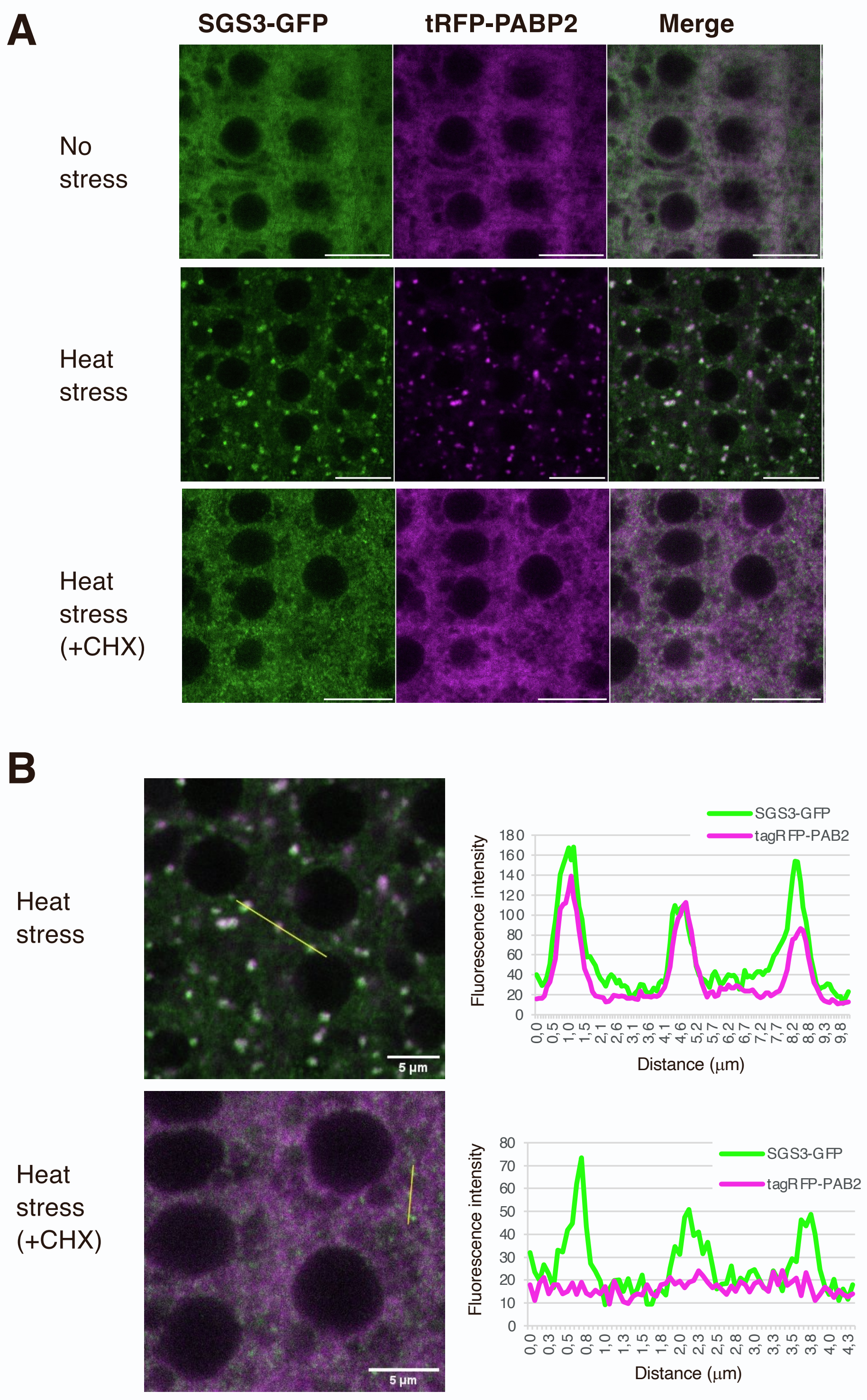

Fig. S3 Cycloheximide inhibits stress granule formation, but not small SGS3 foci.

(**A**) CLSM imaging of 5 day old Arabidopsis pRDR6:SGS3-GFP x pPABP2:tRFP-PABP2 root tip cells before HS (upper panels), after 37°C HS of 30 minutes (middle panels) and after 37°C HS of 30 minutes in the presence of 100µM CHX). Experiment performed in duplicate with three roots per replicate. Bar = 10 µm. Related to Fig. S2B and Fig.2 and see also Table S1.

(**B**) Signal intensity distribution of the total amount of pixels at the x axis shown in the CHX-untreated and CHX-treated cells after 30 minutes of HS at 37°C shown in A. Scale bar = 5 µm. Three independent roots were analyzed. For each of these roots, a selected area of a square of 15 µm side was defined in which 3 different measurements of PCC were performed. Data are represented as mean +/- SEM. For non CHX-treated plants (PC= 0.84 ± 0.01) and for CHX-treated plants (PC= 0.26 ± 0.05).

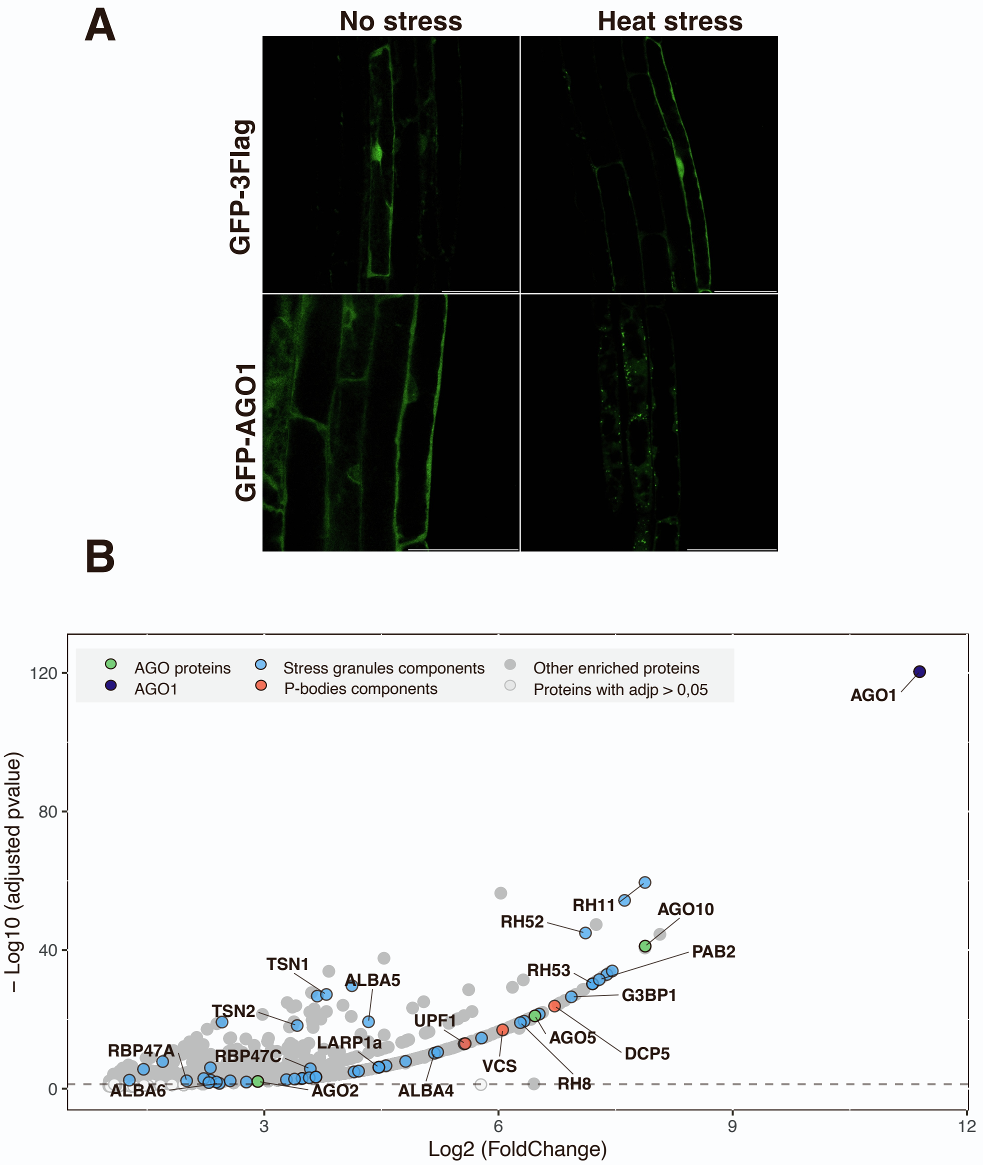

Fig. S4 AGO1 interactome revealed by immunoprecipitation and mass spectrometry

(**A**) CLSM imaging of 7 day old Arabidopsis root tip cells of pAGO1:GFP-AGO1 and p35S:GFP-3Flag transgenic lines before and after 37°C HS of 1 hr. Bar = 50 µm Objective 40X, oil immersion.

(**B**) Volcano plot showing the enrichment during HS of proteins co-purified with the GFP-AGO1 bait compared with GFP alone as control. We compared 9 samples (pAGO1:GFP-AGO1 *ago1-27*) from 3 independent biological replicates to 9 control samples p35S:GFP-3Flag seedlings subjected to 1 hr HS at 37°C. The y and x axes display log2 values from adjusted p values and fold changes, respectively. The horizontal dashed line indicates the threshold above which proteins are significantly enriched (adjusted p values < 0.05). Only AGO1-enriched proteins (log2FC > 1) are shown. Three color-coded functional clusters are highlighted. Enriched proteins are dark grey, cytoplasmic RNA granules related proteins are highlighted in blue, AGO proteins are in green, selected P-bodies markers are in red. The source data are available in Table S2. Related to Fig. 3A.

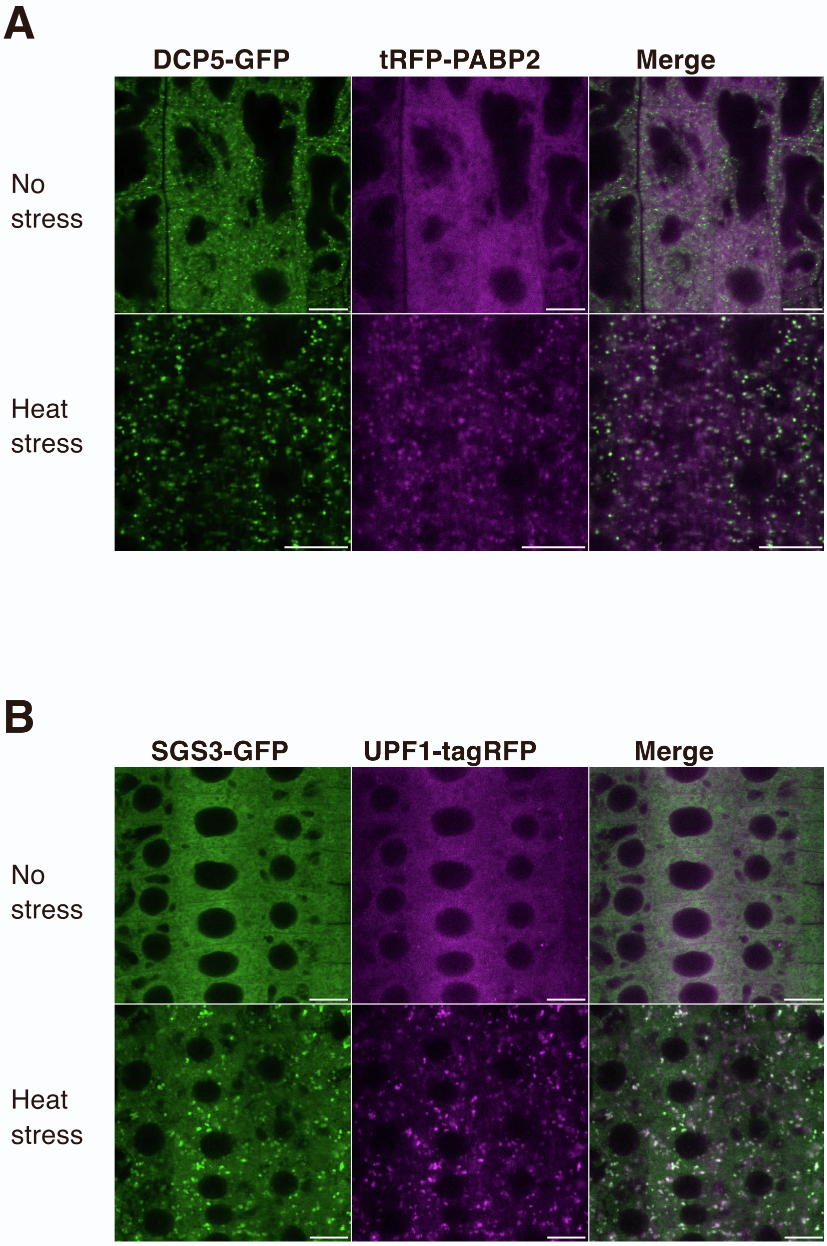

Fig. S5 DCP5/PAB2 and SGS3/UPF1 colocalization during HS.

(A) DCP5 colocalizes with PAB2 in foci under heat-stressed conditions. Confocal laser scanning microscopy on 5 day old Arabidopsis pUB10:DCP5g-GFP *dcp5-1* x pPABP2:tRFP-PABP2 root tip cells before and after 37°C HS of 30 minutes. Experiments were performed in triplicate with three roots per replicate. Data are represented as mean +/- SME. Bar = 10 μm. See also Table S1 and related to Fig. 3B.

Foci number after HS for DCP5-GFP = 479 ± 45; PCC= 0.67 ± 0.04

Foci number after HS for tRFP-PABP2 = 400 ± 13; PCC= 0.61 ± 0.04

(B) SGS3 colocalizes with UPF1 in foci under heat-stressed conditions. Confocal laser scanning microscopy on 5 day old Arabidopsis pRDR6:SGS3-GFP x pUPF1:UPF1g-tagRFP *upf1-5* root tip cells before and after 37°C HS of 30 minutes. Experiments were performed in triplicate with three roots per replicate. Data are represented as mean +/- SME. Bar = 10 μm. See also Table S1 and related to Fig. 3C.

Foci number after HS for SGS3-GFP = 1498 ± 226; PCC= 0.77 ± 0.01

Foci number after HS for UPF1-tagRFP= 1602 ± 217; PCC= 0.77 ± 0.02

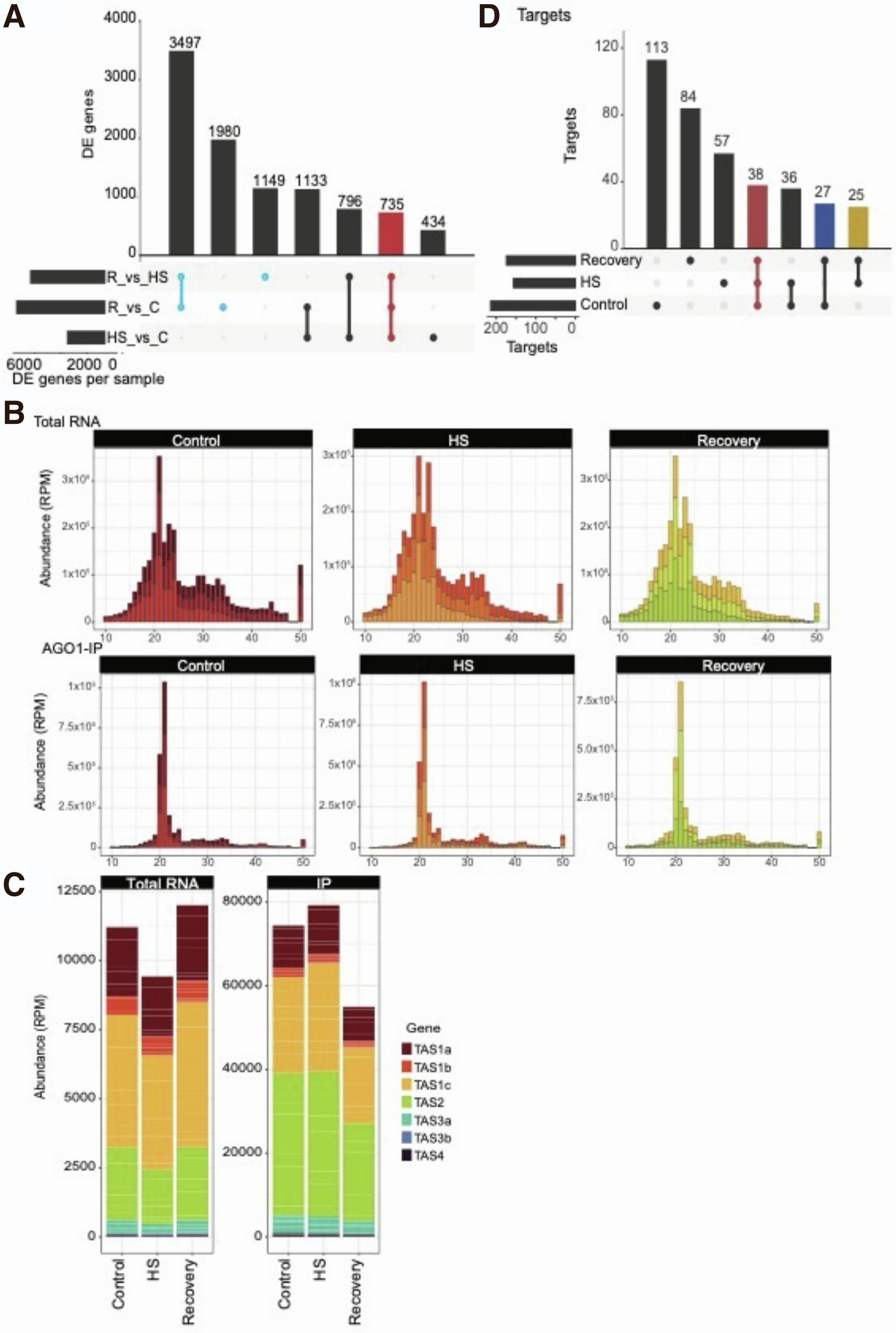

Fig. S6 Differentially expressed coding genes and sRNA accumulation during HS and recovery

(**A**) Upset plot representing statistically significant differentially expressed (DE) genes between all three comparisons . See also Fig. 4A.

(**B**) Size distribution of sRNAs mapping to the Arabidopsis genome (TAIR version 10). The abundance of each size class was normalized to reads per million (RPM) for each library. The x-axis indicates the sRNA size and the y-axis its abundance in RPM-mapped reads. Shown are data from three independent biological replicates, for total RNA (upper panel) and AGO1-IP (lower panel). Related to Fig. 4B.

(**C**) Abundance of each TAS gene in Total RNA (left panel) and AGO1-IP (right panel). The x-axis represents the sample type, and the y-axis represents the abundance of sRNAs mapping each TAS gene. The three biological replicates for each treatment are plotted and stacked together. Each TAS gene is represented with a different color.

(**D**) Upset plot representing the miRNA targets identified by nanoPARE sequencing in each of the treatments. For each upset plot, the bottom left shows the number of differentially expressed genes in each comparison as a horizontal histogram, the bottom right shows the intersection matrix and the upper right shows the size of each combination as a vertical histogram. The red line marks differentially expressed genes common to all three comparisons, the blue lines mark differentially expressed genes during the recovery process. Related to Fig. 4D.

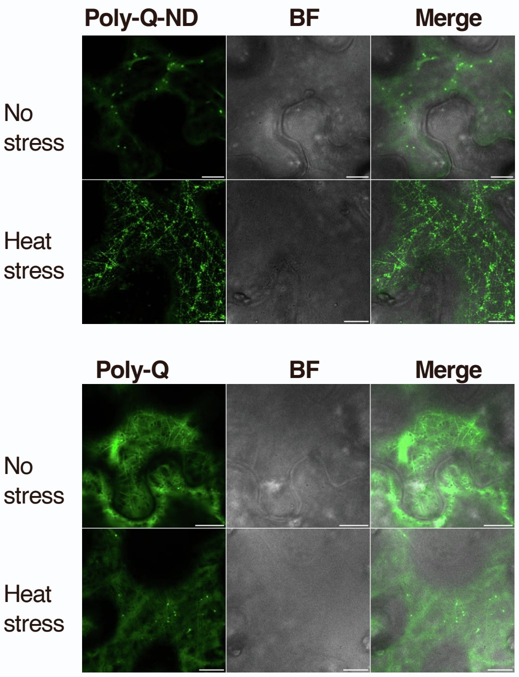

Fig. S7 The Poly-Q domain alone undergoes LLPS in plant cells under HS.
Representative CLSM imaging of *N. benthamiana* leaf epidermal cells transiently expressing GFP-Poly-Q-ND and GFP-Poly-Q proteins in the absence or presence of HS (30 minutes at 37°C). Bar = 10 μm. Objective 40X, oil immersion. BF, bright field. Related to Fig. 5A and Fig. 5C.

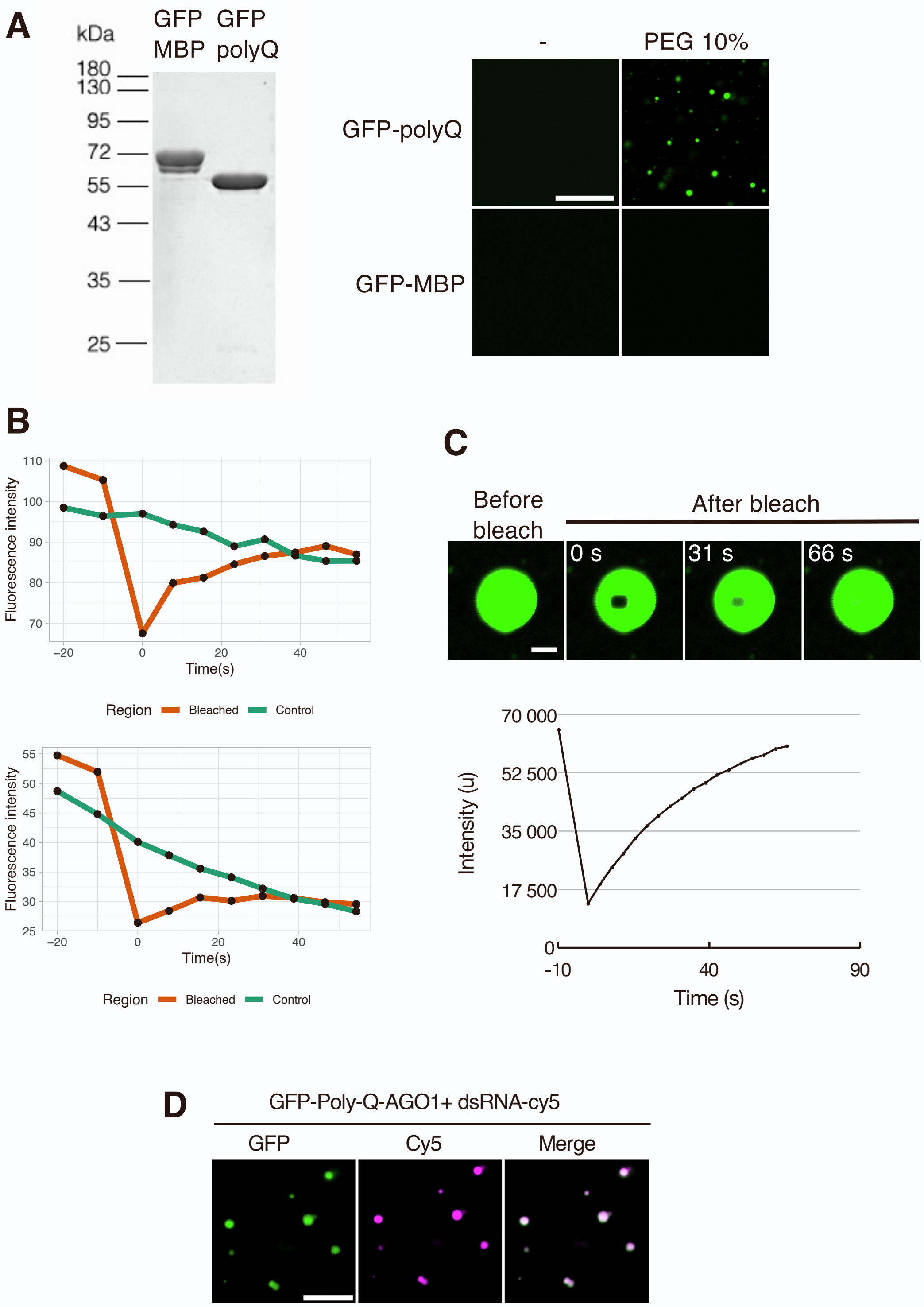

Fig. S8 The Poly-Q domain of AGO1 alone undergoes LLPS and colocalizes with RNA *in vitro*

(A) The Poly-Q domain of AGO1, but not maltose binding protein fused to GFP form droplets *in vitro*. Left panel: SDS-PAGE analysis of the recombinant proteins GFP-polyQ and GFP-MBP used in *in vitro* LLPS assays and EMSA assays. 2,5ug per lane of 12% Tris-glycine SDS-PAGE. Right panel: *In vitro* analysis of droplet formation by recombinant GFP-polyQ protein and control protein GFP-MBP protein at 200mM NaCl concentration in 25mM HEPES pH7.5, 10% PEG 8000. Protein concentration is 4μM. Bar = 20μm. Objective 20X. Related to Fig. 5D.

(B) Two more biological replicates of FRAP assays of pAGO1:GFP-AGO1 *ago1-27* Arabidopsis root tip cells subjected to HS as shown in Fig. 5E.

(C) *In vitro* FRAP analysis of GFP-Poly-Q droplets. Time 0 is set at the photobleaching pulse. Data are representative of four independents experiments. Bar = 2μm. At the bottom, recovery data corresponding to images displayed in the upper part. Related to Fig. 5E.

(D) Colocalization of GFP-Poly-Q protein with Cy5 labelled-dsRNAs upon droplet formation in 25mM HEPES buffer pH7.5, 200mM NaCl, 10% PEG8000. Protein and RNAs are at 4μM. Bar = 10μm. Objective 40X. Related to Fig. 5G.

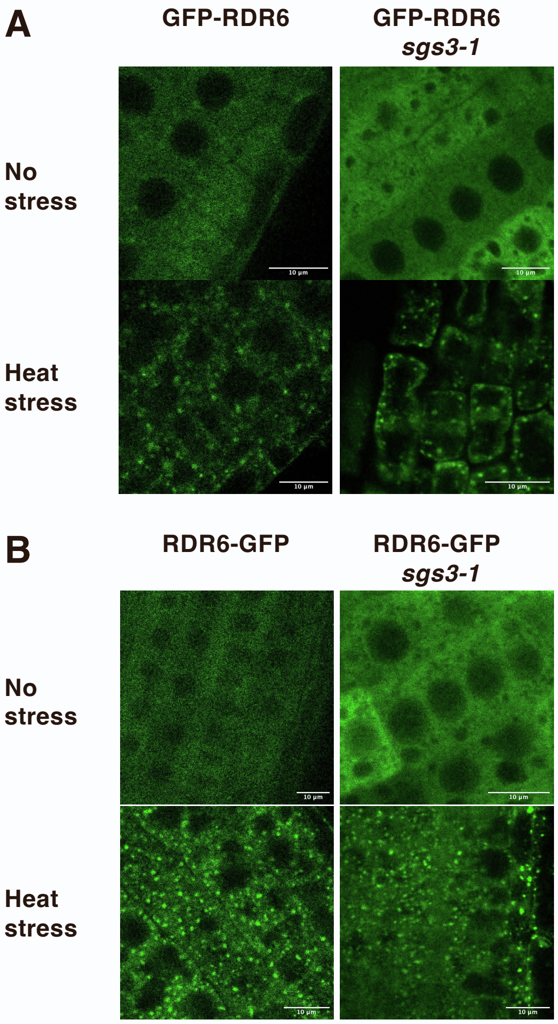

Fig. S9 HS promotes GFP-RDR6 foci formation even in the absence of *SGS3*

(A) Representative CLSM imaging of 5 day old Arabidopsis root tip cells of the p35S:GFP-RDR6 in Col-0 or *sgs3-1* transgenic line subjected to 60 min HS at 37°C. Bar = 10 µm. Objective 60X, water immersion.

(B) Representative CLSM imaging of 5 day old Arabidopsis root tip cells of the p35S:RDR6-GFP in Col-0 or *sgs3-1* transgenic line subjected to 60 min HS at 37°C. Bar = 10 µm. Objective 60X, water immersion.

Related to Fig. 5H.

Lines	Subcellular localization	References
pAGO1:GFP-AGO1(cs) *ago1-36*	Nucleus/cytosol/ER	[1]
pAGO1:GFP-AGO1 *ago1-27*	Nucleus/cytosol/ER	[2]
pAGO1:mCherry-AGO1	Nucleus/cytosol/ER	[2]
pUB10:mCherry-RabG3f (R5)	MVB	[3]
pUB10:mCherry-Rha1/RabF2a (R7)	MVB, tonoplast	[3]
pUB10:mCherry-VTI12 (R13)	TGN/EE	[3]
pUB10:mCherry-SYP22 (R22)	Golgi apparatus	[3]
pRDR6:SGS3-GFP	siRNA bodies	This manuscript
p35S:RDR6-GFP *sgs3-1*	siRNA bodies	[4]
p35S:GFP-3Flag	Nucleus/cytosol	This manuscript
pDCP1:DCP1-YFP *dcp1-3*	P-Bodies	[5]
pUB10:DCP5g-GFP *dcp5-1*	Cytosol/P-Bodies	[5]
pUPF1:UPF1g-tagRFP *upf1-5*	Cytosol/P-Bodies	[5]
pPABP2-tRFP-PABP2	Cytosol/Stress granules	[6]

Table S1 List of Arabidopsis transgenic lines used in this study. Related to Fig. 4 and Fig. S6.

GENOTYPING PRIMERS			
sgs3.14	sgs3-14 LP	AAATTTGGAGTCCAGAATCGG	Genotyping sgs3-14 SALK_001394
	sgs3-14 RP	CAAAGCATCGGAATCATTCTC	Genotyping sgs3-14 SALK_001394
ago1-27	ago1.27_CAPS_F	CCTGGTGAGACAAGTTTGGATC	Genotyping ago1-27 BanII
	ago1.27_CAPS_R	GGGTGAATCAACTCAGCAGTAGAAC	Genotyping ago1-27
NORTHERN PROBES			
ath-miR168		TTCCCGACCTGCACCAAGCGA	Oligo probe for LMW Northern blot
ath-miR398		AAGGGGTGACCTGAGAACACA	Oligo probe for LMW Northern blot
ath-miR403		CGACTTTGTGCGTGAATCTAA	Oligo probe for LMW Northern blot
ath-U6		AGGGGCCATGCTAATCTTCTC	Oligo probe for LMW Northern blot
RT-QPCR			
HSFA2 F	AT2G26150 FWD Set 3	GAA ACG GAG GCT TAC TTC TAC TC	qPCR primers
HSFA2 R	AT2G26150 REV Set 3	CTG CAG CGA ACA ACA TTT CC	qPCR primers
AGO1-LP	AT1G48410	CGGTGGACAGAAGTGGGAAT	qPCR primers
AGO1-RP	AT1G48410	GGTCGAGAAGTGCCCTGAAT	qPCR primers
EXP-LP	At4g26410	GAGCTGAAGTGGCTTCAATGAC	qPCR primers
EXP-RP	At4g26411	GGTCCGACATACCCATGATCC	qPCR primers
TIP41-LP	AT4G34270	GTGAAAACTGTTGGAGAGAAGCAA	qPCR primers
TIP41-RP	AT4G34271	TCAACTGGATACCCTTTCGCA	qPCR primers
CLONING PRIMERS			
pENTRY-Poly-Q-AGO1	AGO1 Poly-Q-fwd	GGGGACAAGTTTGTACAAAAAAGCA<br>GGCTCCATGGTGAGAAAGAGAAGAA<br>CG	cloning of Poly-Q of AGO1 in pDONR221
	AGO1 Poly-Q-rev	GGGGACCACTTTGTACAAGAAAGCT<br>GGGTTTCAATCCTTATCAGGCAGTTC<br>AGCA	
pENTRY-R2-AGO1gUTR-L3	attB2R_AGO1g_F (MC145)	GGGGACAGCTTTCTTGTACAAAGTG<br>GCAATGGTGAGAAAGAGAAGAACGG	cloning of AGO1 full genomic in pDONRP2RP3
	attB3_AGO1gUTR_R (MC146)	GGGGACAACTTTGTATAATAAAGTTG<br>CAGTTAAAGAAAGGATCAAAGTCTGT<br>T	
pENTRY-R2-PolyQ-ND-L3	attB2R_AGO1g_F	GGGGACAGCTTTCTTGTACAAAGTG<br>GCAATGGTGAGAAAGAGAAGAACGG	cloning of PolyQ-ND of AGO1 in pDONRP2RP3
	attB3NterAGO1-300_rev	GGGGACAACTTTGTATAATAAAGTTT<br>CACAGATCAGCACGTGCAAC	
pRDR6:SGS3g-GFP	proSGS2F-HIII	AAGCTTGCAAACCTGTCATATCTGTG<br>G	cloning of RDR6 promoter in pGWB4
	proSGS2R-HIII	AAGCTTTTCTCCTGAAAAAGAAACAT<br>AC	
	attB2SGS3F	GGGGACAAGTTTGTACAAAAAAGCA<br>GGCTTAATGAGTTCTAGGGCTGGTC<br>GGG	cloning of SGS3 in the modified pGWB4 containing RDR6 promoter
	attB2SGS3R	GACCACTTTGTACAAGAAAGCTGGG<br>TAATCATCTTCATTGTGAAGGC	
GFP-AGO1Poly-Q domain-6xHis	AGO int R	GCGTCTCAGCCAGAGCCTTCACCT	cloning of GFP-AGO1Poly-Q domain-6xHis
	AGO1int F	GCGTCTCATGGCTCTCGTGAAGC	
	AGO1-pQ B4 F	GCCGTCTCGCTCGAGGTATGGTGAG<br>AAAGAGAAGAACG	
	AGO1-pQ B4 R	GCCGTCTCGCTCACGAATCCTTATCA<br>GGCAGTTCAGCA	
p35S:GFP-3Flag	612PacAscF	AGCTATTAATTAACCATGGGGCGCG<br>CC	modificatin of the multiple cloning site of the Pily vector by insertion of annealed oligos to create Pily-PA-7Ha
	612PacAscR	GGCGCGCCCCATGGTTAATTAAT	
	612FlagF	CTAGAGGAGATTACAAGGATGATGA<br>CGATAAGGCAGGAGATTACAAGGAT<br>GATGACGATAAGGCAGGAGATTACA<br>AGGATGATGACGATAAGTGAG	cloning of 3Flag sequence by using annealed oligonucleotides and cloning into Pily-PA-7Ha to obtain Pily-PA-3xFlag
	612FlagR	AATTCTCACTTATCGTCATCATCCTT<br>GTAATCCCTGCCTTATCGTCATCATC<br>CTTGTAATCTCCTGCCTTATCGTCAT<br>CATCCTTGTAATCTCCT	
	eGFPNheIF	GCTAGCGGCGTGAGCAAGGGCGAG<br>GAGCTGTTCA	cloning of eGFP sequence in Pily-PA-3xFlag to create p35S:eGFP-3xFlag
	eGFPNheIR	GCTAGCAGCACCTCCCTTGTACAGC<br>TCGTCCATGCCGAGA	
ssRNA and dsRNA GFP sequence preparation	A349_IVTGFP118bp_T7pro_fw	GAAATTAATACGACTCACTATAGGGA<br>TAATGTAAACGGCCACAAGTTCAG	ssRNA and dsRNA GFP substrate preparation
	A347_IVTGFP118bp_rv	GGCCAGGGCACGGGCAG	
	A346_IVTGFP118bp_fw	GTAAACGGCCACAAGTTCAG	
	A348_IVTGFP118bp_T7pro_rv	GTAAACGGCCACAAGTTCAG	
		GAAATTAATACGACTCACTATAGGGA<br>TAATGGCCAGGGCACGGGCAG	

Table S3 List of primers for genotyping, RT-qPCR, cloning and Northern probes. Related to STAR Methods.

		Raw reads	Clean reads		Mapping reads	
RNAseq	Control Rep 1	28785784			18966953	65,89
	Control Rep 2	18884030			16240266	86
	Control Rep 3	17617171			15104962	85,74
	Heat Shock Rep 1	25285035			18688169	73,91
	Heat Shock Rep 2	16111873			13372855	83
	Heat Shock Rep 3	15690800			14007177	89,27
	Recovery Rep 1	21212133			16066070	75,74
	Recovery Rep 2	14122783			11969059	84,75
	Recovery Rep 3	18118377			15214001	83,97
small RNAseq Total RNA	Control Rep 1	21081929	20725739	98,31	16248140	77,07
	Control Rep 2	35352189	33937811	96,00	25054967	70,87
	Control Rep 3	19663490	18831492	95,77	15795316	80,33
	Heat Shock Rep 1	29623804	29259969	98,77	25209609	85,10
	Heat Shock Rep 2	27385129	26161239	95,53	22211581	81,11
	Heat Shock Rep 3	20156616	19271380	95,61	17121616	84,94
	Recovery Rep 1	19928665	19425683	97,48	14580302	73,16
	Recovery Rep 2	22079561	21531257	97,52	17480233	79,17
	Recovery Rep 3	17345155	16344099	94,23	14050128	81,00
small RNAseq AGO1-IP	Control Rep 1	15981581	15346379	96,03	12596123	78,82
	Control Rep 2	9650259	9205516	95,39	6806585	70,53
	Control Rep 3	7004906	6917808	98,76	5812376	82,98
	Heat Shock Rep 1	13867414	13716810	98,91	11757092	84,78
	Heat Shock Rep 2	14109532	13556181	96,08	9358801	66,33
	Heat Shock Rep 3	3143624	3032783	96,47	2461577	78,30
	Recovery Rep 1	11073787	10197218	92,08	7766802	70,14
	Recovery Rep 2	10009882	9662175	96,53	8043758	80,36
	Recovery Rep 3	9162062	8942838	97,61	7556160	82,47
nanoPARE	Control Rep 1	11290888				
	Control Rep 2	12303757				
	Control Rep 3	12716356				
	Heat Shock Rep 1	14079315				
	Heat Shock Rep 2	18091852				
	Heat Shock Rep 3	12246794				
	Recovery Rep 1	16741564				
	Recovery Rep 2	16278404				
	Recovery Rep 3	18846159				

Table S4 Summary table for all sequencing experiments. Related to Fig 4 and Fig. S6.

References

[1] Derrien, B., Baumberger, N., Schepetilnikov, M., Viotti, C., De Cillia, J., Ziegler-Graff, V., Isono, E., Schumacher, K., and Genschik, P. (2012). Degradation of the antiviral component ARGONAUTE1 by the autophagy pathway. Proc. Natl. Acad. Sci. 109, 15942–15946. 10.1073/pnas.1209487109.

[2] Bologna, N.G., Iselin, R., Abriata, L.A., Sarazin, A., Pumplin, N., Jay, F., Grentzinger, T., Dal Peraro, M., and Voinnet, O. (2018). Nucleo-cytosolic Shuttling of ARGONAUTE1 Prompts a Revised Model of the Plant MicroRNA Pathway. Mol. Cell 69, 709-719.e5. 10.1016/j.molcel.2018.01.007.

[3] Geldner, N., Dénervaud-Tendon, V., Hyman, D.L., Mayer, U., Stierhof, Y.D., and Chory, J. (2009). Rapid, combinatorial analysis of membrane compartments in intact plants with a multicolor marker set. Plant J. 59, 169–178. 10.1111/j.1365-313X.2009.03851.x.

[4] Moreno, A.B., De Alba, A.E.M., Bardou, F., Crespi, M.D., Vaucheret, H., Maizel, A., and Mallory, A.C. (2013). Cytoplasmic and nuclear quality control and turnover of single-stranded RNA modulate post-transcriptional gene silencing in plants. Nucleic Acids Res. 41, 4699–4708. 10.1093/nar/gkt152.

[5] Chicois, C., Scheer, H., Garcia, S., Zuber, H., Mutterer, J., Chicher, J., Hammann, P., Gagliardi, D., and Garcia, D. (2018). The UPF1 interactome reveals interaction networks between RNA degradation and translation repression factors in Arabidopsis. Plant J. 96, 119–132. 10.1111/tpj.14022.

[6] Merret, R., Carpentier, M.C., Favory, J.J., Picart, C., Descombin, J., Bousquet-Antonelli, C., Tillard, P., Lejay, L., Deragon, J.M., and Charng, Y.Y. (2017). Heat shock protein HSP101 affects the release of ribosomal protein mRNAs for recovery after heat shock. Plant Physiol. 174, 1216–1225. 10.1104/pp.17.00269.
